# Association between homologous recombination deficiency and outcomes with platinum and platinum-free chemotherapy in patients with triple-negative breast cancer

**DOI:** 10.20892/j.issn.2095-3941.2022.0525

**Published:** 2023-03-02

**Authors:** Yimeng Chen, Xue Wang, Feng Du, Jian Yue, Yiran Si, Xiaochen Zhao, Lina Cui, Bei Zhang, Ting Bei, Binghe Xu, Peng Yuan

**Affiliations:** 1 Department of Medical Oncology and Clinical Trial Center, National Cancer Center/National Clinical Research Center for Cancer/Cancer Hospital, Chinese Academy of Medical Sciences and Peking Union Medical College, Beijing 100021, China; 2 Department of VIP Medical Services, National Cancer Center/National Clinical Research Center for Cancer/Cancer Hospital, Chinese Academy of Medical Sciences and Peking Union Medical College, Beijing 100021, China; 3 The Medical Department, 3D Medicines Inc., Shanghai 201114, China

**Keywords:** Homologous recombination deficiency, triple-negative breast cancer, platinum, survival, *BRCA*

## Abstract

**Objective:**

The choice of chemotherapeutic regimen for triple-negative breast cancer (TNBC) remains controversial. Homologous recombination deficiency (HRD) has attracted increasing attention in informing chemotherapy treatment. This study was aimed at investigating the feasibility of HRD as a clinically actionable biomarker for platinum-containing and platinum-free therapy.

**Methods:**

Chinese patients with TNBC who received chemotherapy between May 1, 2008 and March 31, 2020 were retrospectively analyzed with a customized 3D-HRD panel. HRD positivity was defined by an HRD score ≥ 30 or deleterious *BRCA1/2* mutation. A total of 386 chemotherapy-treated patients with TNBC were screened from a surgical cohort (NCT01150513) and a metastatic cohort, and 189 patients with available clinical and tumor sequencing data were included.

**Results:**

In the entire cohort, 49.2% (93/189) of patients were identified as HRD positive (40 with deleterious *BRCA1/2* mutations and 53 with *BRCA1/2* intact with an HRD score of ≥ 30). In the first-line metastatic setting, platinum therapy was associated with longer median progression-free survival (mPFS) than platinum-free therapy [9.1 *vs.* 3.0 months; hazard ratio (HR), 0.43; 95% confidence interval 0.22–0.84; *P* = 0.01]. Among HRD-positive patients, the mPFS was significantly longer in those treated with platinum rather than platinum-free therapy (13.6 *vs.* 2.0 months; HR, 0.11; *P* = 0.001). Among patients administered a platinum-free regimen, HRD-negative patients showed a PFS significantly superior to that of HRD-positive patients (*P* = 0.02; treatment-biomarker *P*-interaction = 0.001). Similar results were observed in the *BRCA1/2*-intact subset. In the adjuvant setting, HRD-positive patients tended to benefit more from platinum chemotherapy than from platinum-free chemotherapy (*P* = 0.05, *P*-interaction = 0.02).

**Conclusions:**

HRD characterization may guide decision-making regarding the use of platinum treatment in patients with TNBC in both adjuvant and metastatic settings.

## Introduction

Triple-negative breast cancer (TNBC) is a tumor type that does not express estrogen receptor, progesterone receptor, or human epidermal growth factor receptor 2 (HER2)^1–3^. Effective therapeutic strategies are lacking for TNBC^4^. In recent years, immune checkpoint inhibitors and anti-angiogenic drugs have shown efficacy in TNBC treatment^5–8^. However, chemotherapy remains the main treatment for TNBC^9^, and the optimum chemotherapeutic regimen has remained undefined, partially because of the high degree of TNBC heterogeneity. Platinum-based chemotherapy, a first-line treatment option for advanced TNBC, and the neoadjuvant regimen for resectable TNBC^9^ have shown modest advantages over platinum-free regimens. A considerable proportion of patients do not respond to platinum^10–18^. Biomarkers are needed to inform patient selection and allow effective therapy to be provided to defined responding patients while enabling nonresponders to avoid the severe toxicity of ineffective chemotherapeutic regimens.

Previous studies have shown that patients with *BRCA* mutations are platinum-sensitive^19–21^; on this basis, *BRCA* mutations have been clinically used as predictors of platinum therapy efficacy. *BRCA1/2* mutation-associated-signatures overlap with abnormalities in homologous recombination repair (HRR) genes, which are deficient in a substantial subset of TNBC termed sporadic basal TNBC^22,23^. The hallmark aberrations in the *BRCA* and HR gene pathways are sensitive to DNA crosslinking induced by platinum^24–28^. Therefore, studies have been conducted to develop measures including HR deficiency (HRD) detection for identifying both *BRCA1/2*-intact and *BRCA1/2*-deleterious HR-deficient patients, and predicting sensitivity to platinum^29–31^. The Myriad HRD assay was established to assess HRD by measuring loss of heterozygosity (LOH), telomeric allelic imbalance (TAI), and large-scale state transition (LST)^29,32–34^. This assay was initially approved as a companion diagnostic test to identify patients with HRD-positive advanced ovarian cancer for niraparib treatment^35^. In patients with resectable TNBC, the Myriad-HRD score is associated with the response to platinum neoadjuvant therapy^10,36–42^, whereas its predictive value for adjuvant therapy, a controversial platinum treatment for TNBC, remains unknown^9,43,44^. Patients with recurrent or metastatic TNBC experience a similar plight. An observational study has reported that patients with advanced breast cancer treated with platinum and with HRDetect scores ≥ 0.7 have greater 3-month overall survival, thus suggesting the value of HRD in guiding platinum treatment of advanced breast cancer^45^. The TNT trial has compared the response between carboplatin and docetaxel, and biomarker subgroup analyses have revealed that among the Myriad-HRD status, *BRCA* mutation, *BRCA* mRNA level, *BRCA1* promoter DNA methylation^15^, and structural chromosomal instability (CIN)^30^, only *BRCA* mutation and CIN predict a greater benefit from carboplatin over docetaxel in patients with metastatic TNBC, thus indicating a need for optimizing the HRD detection method. The question of whether platinum is an optimum treatment option for *BRCA1/2*-intact patients with HRD remains to be answered.

In this study, we analyzed the association between HRD status and the response to platinum-based treatment compared with platinum-free chemotherapy in patients with advanced TNBC in the first-line setting, and in patients with resectable TNBC in the adjuvant setting. Our aim was to explore the potential of HRD to guide personalized chemotherapy. This study used a Chinese population-based 3D-HRD detection assay incorporating LOH, TAI, and LST with next-generation sequencing (NGS). The associations of HRD with genomic aberrations and clinicopathological features were also explored.

## Materials and methods

### Study design and participants

This study analyzed HRD in Chinese patients with TNBC who received chemotherapy between May 1, 2008 and February 9, 2021 at the National Cancer Center. The study design and sample used during HRD algorithm development and the exploration of HRD’s clinical relevance in each step are shown in **Supplementary Figure S1**. For the assessment of HRD’s clinical relevance, 386 chemotherapy-treated patients with TNBC were screened from a surgical cohort (NCT01150513)^46^ and a metastatic cohort^11,14,47^ administered chemotherapy in the adjuvant and first-line metastatic setting, respectively. In the metastatic cohort, 495 patients with metastatic TNBC were screened from more than 10,000 patients with breast cancer, thus resulting in the selection of 40 patients with qualified samples. Finally, 189 patients with TNBC with available clinical and tumor sequencing data were included in this study. All tumor samples were collected during surgical operation and were subjected to NGS to evaluate HRD and genomic mutations. The primary aim of this study was to explore the association between HRD status and clinical outcomes. The associations of HRD with genomic mutations and clinicopathological features were also investigated. Data were analyzed from July 1 to September 1, 2021. As of March 22, 2021, the median follow-up times of the metastatic and adjuvant cohorts were 27.7 [interquartile range (IQR): 15.9–44.5] and 86.8 (IQR: 75.8–101.7) months, respectively. This study was reviewed and approved by the ethics committee of the Cancer Hospital, Chinese Academy of Medical Sciences and Peking Union Medical College (Approval No. 19/147-1931) and was conducted in accordance with the Declaration of Helsinki (as revised in 2013). Exemption from written informed consent was approved by the above ethics committee. This study is presented according to the STROBE reporting checklist.

### NGS and 3D-HRD algorithm development

NGS and HRD detection were conducted at 3D Medicines, Inc. (Shanghai, China), a College of American Pathologists-accredited and Clinical Laboratory Improvement Amendments-certified laboratory. Genomic DNA was extracted from formalin-fixed paraffin-embedded tumor and paired normal tissue samples. Eligible tumor tissue with ≥ 20% tumor cells was retained for subsequent analyses. Indexed libraries were subjected to probe-based hybridization with a customized NGS panel targeting 733 cancer-associated genes (**Supplementary Table S1**), including HRR-associated genes. The 3D-HRD algorithm was developed to estimate HRD with a training cohort of 594 tissue samples from Chinese patients with breast or ovarian cancer, on the basis of more than 10,000 single-nucleotide polymorphisms (SNPs) in the human genome. The HRD score was calculated as the sum of the LOH, TAI, and LST. All 3 measures predict benefits from neoadjuvant platinum therapy in patients with TNBC^36^. The threshold score for HRD was determined to be 30 to achieve 95% sensitivity in identifying patients with *BRAC1/2*-deficient mutations, through analysis of the HRD status of the training cohort of 106 breast and 488 ovarian tumors with known *BRCA1/2* mutation status^36,48^. *BRCA* deficiency was defined as having pathogenic or likely pathogenic deleterious *BRCA1/2* mutations, with LOH in the wild-type copy. A tumor was defined as HRD-positive if it had an HRD score of ≥ 30 and/or a deleterious mutation in *BRCA1/2*. *BRCA1/2*-intact tumors with an HRD score of < 30 were defined as HRD-negative. Technical validation of the HRD assay was performed with 2 HCC cell lines and 75 tumor samples from an independent cohort of patients with breast or ovarian cancer. The HRD assay exhibited a sensitivity of 95.7% and a limit of detection of ≥ 20% tumor cells in tumor tissue (**Supplementary Methods, Supplementary Figures S2 and S3**).

### Statistical analysis

Continuous variables were compared with Student’s t-test or the Wilcoxon test, and categorical variables were compared with the chi-square test or Fisher’s exact test, as appropriate. Simple linear regression analysis was performed to determine the relationship between the HRD score and the Ki67 proliferation index (%). Survival curves and median survival times for all groups were depicted with Kaplan–Meier survival curves and analyzed with the log-rank test. The hazard ratio (HR) and 95% confidence interval (CI) were estimated with the Cox proportional hazards model. Median follow-up was analyzed with the reverse Kaplan–Meier method. Univariate survival analyses were performed with the Cox proportional-hazards model. Interaction *P* values (*P*-interaction) are reported for the joint effects of biomarkers and treatment. *P* < 0.05 was considered statistically significant. Statistical analyses were performed in R software (version 3.6.1).

## Results

### Correlation between HRD and clinicopathological features

The mean age of the 189 included patients was 48.3 years (standard deviation, 9.5), and all patients were women. The remaining 197 patients were excluded because of incomplete clinical information (*n* = 1), or a lack of sufficient or qualified tumor samples for sequencing (*n* = 196) (no tumor sample, *n* = 159; unqualified tissue sample, *n* = 23; DNA extraction failure, *n* = 4; and sequencing library construction failure, *n* = 10). Among the 189 included patients, 149 patients received platinum-containing or platinum-free chemotherapy in the adjuvant setting (surgical cohort), and 40 patients with metastatic disease received chemotherapy in the first-line metastatic setting. The major pathological type was ductal carcinoma, accounting for 96.3% (182/189) of the patients in the entire cohort. Of the 149 patients in the surgical cohort, more than half (91/149, 61.1%) had stage II disease (**Table 1**).

**Table 1:** Correlation between clinicopathological features and HRD status in 189 triple-negative breast cancers

Characteristics	No. (%)			
	Total (*n* = 189)	HRD+ (*n* = 93)	HRD− (*n* = 96)	*P* value
Age, mean (standard deviation), years	48.3 (9.5)	46.6 (9.0)	50.0 (9.7)	0.01
Menopausal status				0.07
Premenopausal	102 (54.0)	57 (61.3)	45 (46.9)	
Postmenopausal	87 (46.0)	36 (38.7)	51 (53.1)	
Ki67 proliferation index (%)				
Median (range)	60 (5.0, 95.0)	65 (10.0, 95.0)	40 (5.0, 95.0)	< 0.001
N/A	5	3	2	
Tumor stage				0.13
cT1	91 (48.4)	38 (41.3)	53 (55.2)	
cT2	92 (48.9)	52 (56.5)	40 (41.7)	
cT3	5 (2.7)	2 (2.2)	3 (3.1)	
N/A	1	1		
Pathological status				0.15
Ductal	182 (96.3)	87 (93.5)	95 (99.0)	
Medullary	4 (2.1)	3 (3.2)	1 (1.0)	
Others	3 (1.6)	3 (3.2)	0 (0.0)	
Histological grade				0.09
II	52 (29.2)	20 (22.7)	32 (35.6)	
III	126 (70.8)	68 (77.3)	58 (64.4)	
N/A	11	5	6	
Clinical stage				0.15
I	50 (26.5)	22 (23.7)	28 (29.2)	
II	91 (48.1)	52 (55.9)	39 (40.6)	
III	8 (4.23)	2 (2.20)	6 (6.3)	
IV	40 (21.2)	17 (18.7)	23 (24.0)	
PD-L1 IPS expression				0.36
<1%	116 (77.9)	62 (81.6)	54 (74.0)	
≥1%	33 (22.1)	14 (18.4)	19 (26.0)	
N/A	40	17	23	
PD-L1 TPS expression				1.00
<1%	133 (89.3)	68 (89.5)	65 (89.0)	
≥1%	16 (10.7)	8 (10.5)	8 (11.0)	
N/A	40	17	23	
*BRCA 1/2*				< 0.001
Mutant	40 (21.2)	40 (43.0)	0 (0.00)	
Wild-type	149 (78.8)	53 (57.0)	96 (100)	
HRR-associated genes				< 0.001
Mutant	68 (36.0)	49 (52.7)	19 (19.8)	
Wild-type	121 (64.0)	44 (47.3)	77 (80.2)	

HRD, homologous recombination deficiency. A tumor was defined as HRD+ on the basis of HRD scores ≥ 30 and/or deleterious mutation in *BRCA1/2*, whereas *BRCA1/2*-intact tumors with HRD scores < 30 were defined as HRD−. PD-L1, programmed death ligand 1; TPS, tumor proportion score, the proportion of viable tumor cells showing partial or complete membrane PD-L1 staining at any intensity; IPS, immune proportion score (IPS), the percentage of tumor-infiltrating immune cells with PD-L1 staining at any intensity; HRR, homologous recombination repair; N/A, not available.

**Figure 1:**
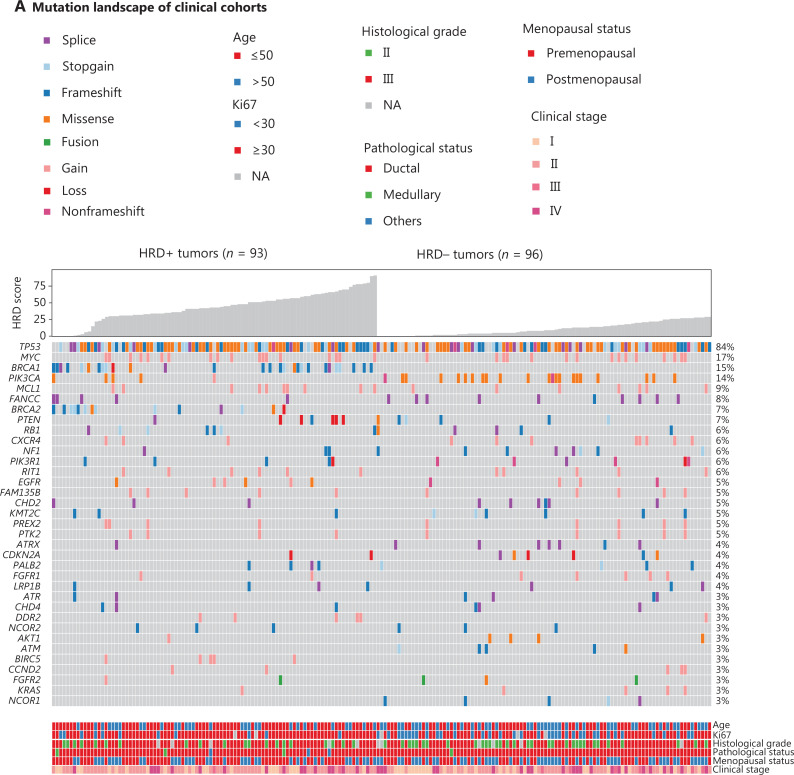

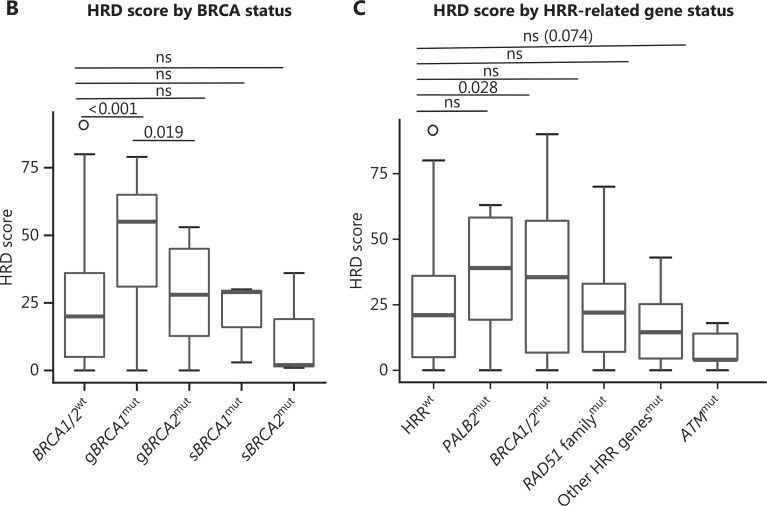
Correlation of HRD with molecular and clinical features. (A) Pathogenic alterations in tumor-associated genes and HRD scores, detected by next-generation sequencing of tumor DNA from 149 patients with TNBC. Patients with an HRD score ≥ 30 and/or deleterious mutations in *BRCA1/2* were classified as HRD-positive, whereas patients with an HRD score < 30 and *BRCA1/2*-intact status were defined as HRD-negative. Mutated genes are listed in descending order from most to least frequently altered. The clinical features include, from top to bottom, patient age, Ki67 expression, disease stage, histologic grade, and pathological subtype. B, HRD scores of *BRCA*-wild-type tumors and tumors with either germline or somatic *BRCA1/2* mutations. C, HRD scores of tumors with HRR gene mutations. HRD scores were determined with 3D-HRD assays. Differences in groups were calculated with either Wilcoxon or t-tests, as appropriate. *P* < 0.05 was considered statistically significant.

Among the entire metastatic and surgical TNBC cohort (*n* = 189), 49.2% (93/189) of patients were classified as HRD-positive. The baseline characteristics indicated younger age (*P* = 0.01) and a higher Ki67 proliferation index (%) (*P* < 0.001) in the HRD-positive patients than the HRD-negative patients (**Table 1**). A high HRD score was positively associated with the levels of Ki67 expression (Spearman’s correlation coefficient, 0.32; *P* < 0.001), histologic grade (grade III *vs.* II: median 27.0 *vs.* 13.0; *P* = 0.02), and T stage (cT2 *vs.* cT1: median 29.0 *vs.* 14.0; *P* = 0.001). The HRD score was not significantly correlated with lymph node status, TNM stage, N stage, menopausal status, or PD-L1 expression (**Figure 1A, Supplementary Figures S4 and S5**).

### Genomic association with HRD

*BRCA1/2* mutations were identified in 21.2% (40/189) of patients from the entire metastatic and surgical TNBC cohort. Forty-three percent (40/93) of HRD-positive patients were classified as having deleterious *BRCA1/2*-mutation, and 57.0% (53/93) were classified as having *BRCA1/2* intact (**Table 1 and Supplementary Table S2**). The median HRD scores of *BRCA*^mut^ and *BRCA*^wt^ patients were 35.5 (range 0–90) and 20.0 (range 0–91), respectively. Among the 40 patients carrying *BRCA*1/2 mutations, 85.0% (34/40) had germline *BRCA1/2* mutations (g*BRCA1/2*), and 27.5% (11/40) had somatic *BRCA1/2* mutations (s*BRCA1/2*). Two patients carried both g*BRCA1* and s*BRCA1*, one patient had concurrent g*BRCA2* and s*BRCA2*, and 2 carried both g*BRCA1* and s*BRCA2* (**Supplementary Figure S6 and Table S2**).

Compared with *BRCA1/2*-intact patients, those carrying *BRCA1/2* mutations had a significantly higher HRD score (median, 35.5 *vs.* 20.0; *P* = 0.02, **Supplementary Figure S7A**). Because the *BRCA1/2* mutation subtypes may exert distinct effects on genomic instability^49^, we further examined HRD status among patients with g*BRCA1*/*2* and s*BRCA1/2*, and found that g*BRCA*^mut^ patients had a numerically higher HRD score (median, 48.0 *vs.* 16.0; *P* = 0.05) than their s*BRCA*^mut^ counterparts (**Supplementary Figures S7B and S8**). Further analysis revealed that only g*BRCA1*^mut^ patients had a significantly higher HRD score than *BRCA1/2*-intact patients (median: 55.0 *vs.* 20.0; *P* < 0.001, **Figure 1B**), thus suggesting a critical role of germline *BRCA1* mutation in genomic instability in TNBC.

Because LOH was incorporated into the algorithm used to calculate the HRD score, we analyzed the HRD scores among *BRCA*^mut^ patients on the basis of the *BRCA* LOH status. *BRCA*^LOH^ was observed in 82.1% of patients with *BRCA1* mutation, compared with 35.7% of *BRCA2*-mutated patients (*P* = 0.005). The HRD score of *BRCA1*^LOH^ patients was significantly higher than that of the *BRCA1*^non-LOH^ population (median: 53.0 *vs.* 1.5, *P* = 0.008). Further analysis revealed that, among the *BRCA1*^LOH^, *BRCA1*^non-LOH^, *BRCA2*^LOH^, and *BRCA2*^non-LOH^ subgroups, the *BRCA1*^LOH^ group achieved the highest HRD score and was the only subgroup showing a significantly higher HRD score than the *BRCA*^wt^ group (*P* < 0.001), thus suggesting that LOH of *BRCA1* had a relatively greater contribution to HRD (**Supplementary Figure S7C and S7D**). Notably, a patient with *gBRCA1* LOH had an HRD score of 90.

Because deficiencies in other HRR-associated genes may also confer genomic instability, we analyzed the association of the HRD score with deleterious variants, defined as pathogenic/likely pathogenic mutations, in 15 HRR-associated genes (**Supplementary Table S3**). Deleterious HRR-associated gene mutations were identified in 36.0% of patients from the entire 189-patient cohort (**Supplementary Figure S9**). Patients with HRR gene mutations had HRD scores similar to those of HRR^wt^ patients (median, 28.0 *vs.* 21.0, *P* = 0.83, **Supplementary Figure S10A and S10B**). Serendipitously, all 5 patients with ATM mutations were HRD-negative and had HRD scores less than 18 (**Figure 1C and Supplementary Figure S10C**).

In addition to HRR gene mutations, deleterious mutations in other cancer-associated genes were investigated for HRD relevance. *PIK3CA* (*P* = 0.001) and *BIRC5* (*P* = 0.03) mutations were strongly associated with HRD status. The *PIK3CA* mutation frequency was 22.9% (22/96) in HRD-negative patients, compared with 5.4% (5/93) in HRD-positive patients. *FANCC* (10.4% *vs.* 5.4%), *NF1* (8.3% *vs.* 3.2%), *ATRX* (7.3% *vs.* 1.1%), and *NCOR1* (5.2% *vs.* 0%) were more commonly mutated in HRD-negative than HRD-positive patients. *BIRC5*, *SOX2*, and *PDCD1LG2* mutations appeared in only HRD-positive patients (**Figure 1A and Supplementary Table S4**). Interestingly, patients with *PIK3CA* mutations was correlated with shorter disease-free survival (DFS) than *PIK3CA*-intact patients in patients treated with platinum-containing adjuvant therapy (HR, 0.25; 95% CI 0.08–0.80; *P* = 0.01, **Supplementary Figure S11**).

### Utility of HRD in guiding first-line chemotherapeutic treatment for metastatic TNBC

We analyzed the clinical outcomes of 40 patients with TNBC with metastatic TNBC treated with first-line platinum-based treatment, compared with patients receiving platinum-free chemotherapy (21 *vs.* 19; mean cycles of treatment, 5 *vs.* 4), to elucidate the utility of HRD in guiding first-line chemotherapeutic treatment for metastatic TNBC. Eight (8/40, 20%) patients had received neoadjuvant chemotherapy (5 patients received 6 cycles, and 3 patients received 8 cycles). The remaining 32 patients had no exposure to cancer drugs before tumor resection. Of the 8 patients treated with neoadjuvant chemotherapy, 5 and 3 were administered platinum and platinum-free drugs, respectively. No patients had prior exposure to radiation before surgery. Thirty-four patients (34/40, 85%) received adjuvant chemotherapy, and 22 (22/40, 55%) were administered radiotherapy postoperatively. At the data cutoff, disease progression or death had occurred in 38 patients. Among the entire metastatic TNBC cohort, 17 patients were HRD-positive, and 23 were HRD-negative. The baseline clinical characteristics were balanced (**Supplementary Table S5**).

**Figure 2:**
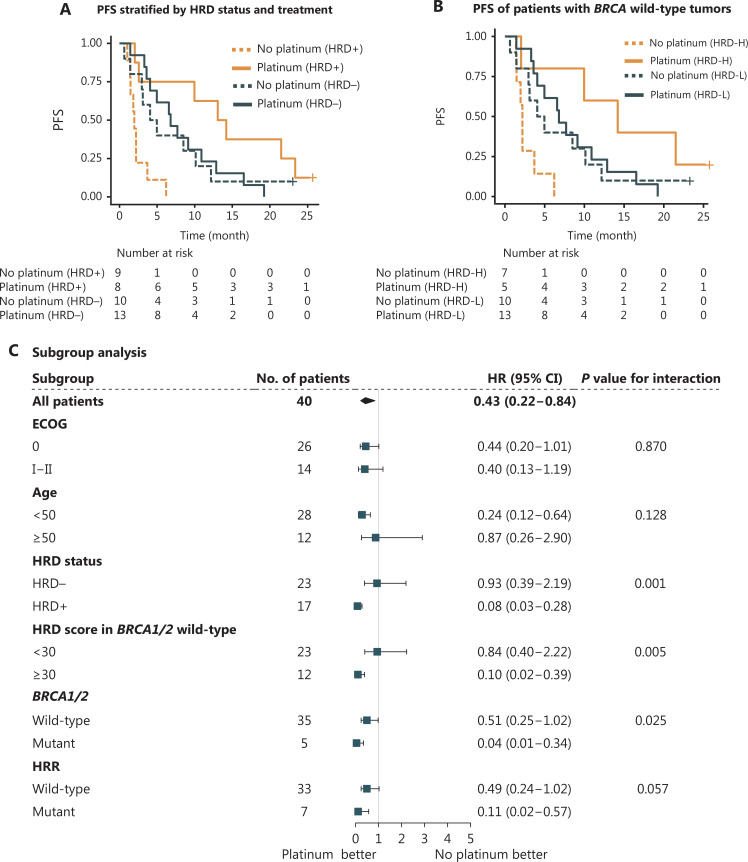
Association of progression-free survival (PFS) with HRD in metastatic TNBC. (A) Kaplan–Meier curves showing the PFS of metastatic patients with TNBC (*n* = 40) in each group, by chemotherapeutic regimen and HRD status. HRD+ was defined by an HRD score ≥ 30 and/or deleterious mutations in *BRCA1/2*, whereas *BRCA1/2*-intact tumors with HRD scores < 30 were defined as HRD−. (B) PFS of *BRCA1/2*-wildtype TNBC treated with first-line platinum-containing or platinum-free chemotherapy (*n* = 35). Patients with HRD scores ≥ 30 were defined as HRD score high (HRD-H), whereas those with an HRD score < 30 were defined as HRD score low (HRD-L). (C) Subgroup analyses according to baseline characteristics including ECOG, age, HRD score, and mutations in *BRCA*1/2 and HRR genes. Forest plots showing the estimated hazard ratios (HR) for progression with a univariable Cox proportional hazards model; 95% confidence intervals (CI) of HRs are represented by horizontal bars. HRR, homologous recombination repair. HRD, homologous recombination deficiency. ECOG, Eastern Cooperative Oncology Group performance score.

The progression-free survival (PFS) of patients receiving platinum-containing treatment was significantly longer than that of patients receiving platinum-free therapy [median PFS (mPFS), 9.1 *vs.* 3.0 months; HR, 0.43; 95% CI 0.22–0.84; *P* = 0.01]. The difference was more significant in HRD-positive patients (platinum *vs.* platinum-free, mPFS, 13.6 *vs.* 2.0 months; HR, 0.11; 95% CI 0.02–0.51; *P = *0.001, **Figure 2A**). For HRD-negative patients, no significant difference in PFS was observed between the platinum and platinum-free groups (mPFS, 6.8 *vs.* 4.5 months; HR, 0.88; 95% CI 0.70–2.10; *P* = 0.77). For patients treated with platinum, a longer PFS was observed in the HRD-positive population than in the HRD-negative population (mPFS, 13.6 *vs.* 6.8 months; HR, 0.35; 95% CI 0.12–1.00; *P* < 0.05). Among patients who received platinum-free therapy, HRD-negative patients experienced a longer PFS than their HRD-positive counterparts (mPFS, 4.5 *vs.* 2.0 months; HR, 0.30; 95% CI 0.10–0.88; *P* = 0.02, **Figure 2A and Supplementary Table S6**). No significant differences in the objective response rate or disease control rate was observed in the unselected group or subgroups stratified by HRD status (**Supplementary Figure S12**).

Consistently, in *BRCA1/2*-intact patients, PFS also favored platinum therapy (mPFS, 8.4 *vs.* 3.1 months; HR, 0.51; 95% CI 0.25–1.00; *P* = 0.05), particularly in HRD score-high patients (HRD score ≥ 30 *vs.* HRD score < 30, mPFS, 14.2 *vs.* 2.2 months; HR, 0.10; 95% CI 0.01–0.84; *P* = 0.01). Among *BRCA1/2*-intact patients treated with platinum, HRD score-high patients had a PFS twice that of HRD score-low patients (mPFS, 14.2 *vs.* 6.8 months; HR, 0.32; 95% CI 0.09–1.20; *P* = 0.07), but in patients administered a platinum-free regimen, HRD score-high was associated with a shorter PFS (mPFS, 2.2 *vs.* 4.5 months; HR, 3.00; 95% CI 0.96–9.10; *P* = 0.05, **Figure 2B**).

Univariable analysis revealed that among age, ECOG score, HRD status, *BRCA* status, and mutation in HRR genes, only HRD status showed a statistically marginally significant association with a PFS benefit in the platinum-containing group (**Supplementary Table S7**). Forest plots indicated a significant benefit from platinum in HRD-positive patients compared with HRD-negative patients (interaction *P* = 0.001), thus indicating that HRD-positive patients may benefit more from platinum (**Figure 2C**). We observed a significantly prolonged PFS from platinum in *BRCA1/2* mutated patients compared with *BRCA1/2*-wildtype patients (interaction *P* = 0.025), thus indicating that *BRCA1/2*-mutated patients may benefit more from platinum therapy.

The median OS was 36.8 and 18.9 months for patients with metastatic TNBC who were treated with platinum and platinum-free chemotherapy, respectively. No difference was observed in overall survival between the treatment groups (**Supplementary Figure S13**).

### Association of HRD with clinical outcomes of patients with TNBC receiving adjuvant chemotherapy

**Figure 3:**
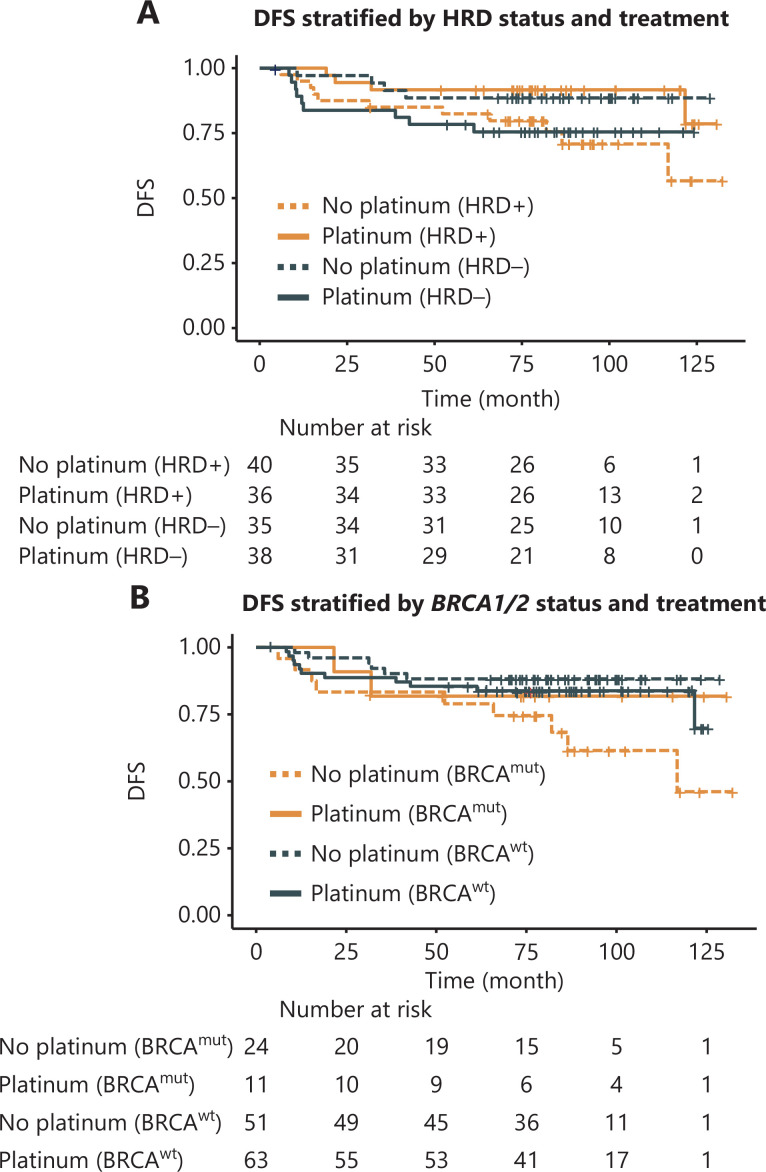
Association of HRD with disease-free survival (DFS) among patients with TNBC receiving adjuvant chemotherapy in the surgical cohort. (A) DFS of HRD+/− patients with TNBC receiving platinum-containing in comparison to platinum-free chemotherapy. HRD+ was defined by an HRD score ≥ 30 and/or deleterious mutations in *BRCA1/2*, whereas *BRCA1/2*-intact tumors with HRD scores < 30 were defined as HRD−. (B) DFS of *BRCA*1/2^mut^ and *BRCA1/2*^wt^ patients with TNBC treated with platinum-containing or platinum-free regimens.

In the surgical cohort of 149 patients with TNBC, no patients received neoadjuvant therapy. After surgery, 74 patients were treated with 6 cycles of TP (docetaxel: 75 mg/m^2^ or paclitaxel 175 mg/m^2^ day 1; carboplatin AUC = 5, day 1), and 75 received 4 cycles of EC (epirubicin: 90 mg/m^2^; cyclophosphamide: 600 mg/m^2^, day 1) followed by 4 cycles of T (docetaxel: 75 mg/m^2^ or paclitaxel 175 mg/m^2^, day 1). Approximately 26.1% (39/149) of patients had received postoperative radiation. At the data cutoff, 28 (18.8%) patients had experienced disease recurrence or died. Seventy-six patients were HRD-positive, and 73 were HRD-negative (**Supplementary Table S8**). The HRD-positive patients in the platinum group tended to benefit more than those in the platinum-free group (HR, 0.33; 95% CI 0.11–1.10; *P* = 0.05) and did not reach mDFS, and the interaction between HRD status and treatment was significant (*P* = 0.02). In addition, for HRD-positive patients, the 5-year DFS rate after treatment with platinum was 91.7%, which was numerically better than the 82.4% value for those treated with platinum-free chemotherapy **(Figure 3A and Supplementary Table S9**). Among the patients treated with platinum-free chemotherapy, no significant difference in DFS was observed between HRD-positive and HRD-negative patients (HR, 0.39; 95% CI 0.12–1.20; *P* = 0.10, **Figure 3A**). Among patients treated with platinum, HRD-positive patients tended to benefit more than HRD-negative patients, but the difference was not significant (HR, 0.35; 95% CI 0.10–1.20; *P* = 0.08, **Supplementary Tables S9 and S10**). *BRCA1/2* mutation (24%, 35/149) was associated with poor prognosis (HR, 2.1; 95% CI 1.0–4.6; *P* < 0.05) in the unstratified surgical cohort but not in the platinum-treated subgroup (**Figure 3B, Supplementary Tables S9 and S10**). In addition, *BRCA*-intact patients exhibited longer DFS than *BRCA1/2*-mutated patients in the platinum-free group (mDFS not reached *vs.* 116.8 months; HR, 0.30; 95% CI 0.11–0.85; *P* = 0.02), whereas no significant difference was observed in the patients treated with platinum (**Figure 3**). In the *BRCA*-intact subset, no difference was found in DFS between the different treatment or HRD status groups (**Supplementary Figure S14**).

## Discussion

This study provides novel evidence of the utility of HRD in guiding chemotherapeutic decision-making in a metastatic cohort and a surgical cohort treated with platinum-containing and platinum-free chemotherapy. Our findings revealed that among HRD-positive patients with metastatic disease, the mPFS was significantly better in patients administered first-line platinum-based treatment than in patients receiving platinum-free treatment (mPFS, 13.6 *vs.* 2.0 months; *P* = 0.001; treatment-biomarker *P*-interaction = 0.001). In patients with metastatic disease who were treated with a first-line platinum-free regimen, HRD-negative status was associated with a longer PFS (mPFS, HRD− *vs.* HRD+, 4.5 *vs.* 2.0 months; *P* = 0.02). However, for patients with metastasis who were treated with first-line platinum regimens, HRD-positive patients benefited more than HRD-negative patients (mPFS 13.6 *vs.* 6.80 months, *P* < 0.05). In the *BRCA1/2*-intact subgroup, PFS also favored platinum therapy in HRD-high patients (mPFS, 14.2 *vs.* 2.2 months; *P* = 0.01), thus informing the first-line platinum treatment choice for metastatic TNBC. In the surgical cohort of patients with TNBC treated with adjuvant chemotherapy, HRD-positive patients treated with platinum tended to experience a greater benefit than those treated with platinum-free therapy (*P* = 0.05, *P*-interaction = 0.02).

Several studies have investigated the efficacy of platinum-containing regimens in treating patients with metastatic TNBC. In a randomized phase II trial comparing first-line docetaxel-cisplatin (TP) and docetaxel-capecitabine (TX) in patients with metastatic TNBC, the mPFS of the TP group was significantly longer than that of the TX group (10.9 *vs.* 4.8 months)^14^. Consistently with these findings, an open-label, multicenter randomized crossover trial (CBCSG006) has indicated significantly better mPFS for the platinum-containing group than the paclitaxel-containing group (7.7 *vs.* 6.1 months)^13^. A multicenter, real-world retrospective study of 495 Chinese patients with metastatic TNBC has also reported a longer PFS elicited by first-line platinum-based chemotherapy than platinum-free chemotherapy (8.4 *vs.* 6.0 months)^11^. Although previous reports have shown that patients with metastatic TNBC may benefit more from platinum than platinum-free therapy, 36.4%–68.6% of patients with TNBC are insensitive to platinum^11,14–16^. In addition, owing to the toxicity-associated adverse effects induced by platinum and the physical limitations of patients with advanced tumors, clinicians remain hesitant in deciding on treatment options, particularly for patients carrying no *BRCA1/2* mutations. Biomarkers guiding treatment are urgently required beyond the context of *BRCA1/2* mutations. In our patients with metastatic TNBC who were treated with first-line platinum and platinum-free chemotherapy, mPFS favored platinum in the HRD-positive group but not in the HRD-negative group, thus showing a significant treatment-biomarker interaction (*P* = 0.001). The results from the *BRCA1/2*-intact subpopulation were consistent with that in the population not stratified by *BRCA1/2* mutations. These results differed from those of the TNT trial, in which no interaction was observed between HRD and the clinical outcomes of patients with metastatic TNBC receiving platinum and platinum-free therapy, according to the report published in 2018^15^. Potential explanations for the difference in results between the TNT trial and our study include that the 2 HRD methods differed (3D-HRD *vs.* myChoice HRD), the study population was diverse (100% Chinese *vs.* 87.2% White/6.1% Black/2.9% Asian/3.5% not stated/0.3% mixed), the onset age differed (mean of 48 y *vs.* 55 y), and the disease status varied (metastatic 100% *vs.* 90.2%). Moreover, another post hoc analysis of the TNT trial reported in 2021 has revealed that patients with TNBC tumors lacking high-level amplifications and displaying intermediate CIN benefit more from carboplatin than docetaxel. These data indicate a complex association of the degree of genomic instability with treatment response in patients with metastatic TNBC^30^.

In contrast to the recommendation of platinum therapy for metastatic TNBC, platinum use in adjuvant therapy remains controversial. In a recent randomized, multicenter phase II noninferiority clinical trial investigating platinum-containing adjuvant chemotherapy in patients with operable TNBC, the activity of taxanes combined with carboplatin chemotherapy was similar to the standard regimen of epirubicin and cyclophosphamide followed by taxanes (5-year DFS, 84.4% *vs.* 85.8%, *P* noninferiority=0.03)^46^. In another retrospective study of platinum efficacy in patients with early stage TNBC, addition of carboplatin to standard adjuvant chemotherapy was not associated with improved relapse-free survival or OS^43^. However, a phase III randomized PATTERN trial has suggested a significantly longer DFS in the platinum treatment group than the platinum-free treatment group (5-year DFS, 86.5% *vs.* 80.3%)^18,44^. Therefore, biomarkers must be screened to predict platinum efficacy in the adjuvant setting. The patients in the surgical TNBC cohort in this study were recruited from our noninferiority phase II trial^46^. The 5-year DFS rate of patients receiving adjuvant platinum therapy was 91.7%, which was numerically better than the value of 82.4% in those receiving platinum-free therapy. Although platinum-containing therapy did not provide a significant benefit in the adjuvant setting, HRD-positive patients treated with platinum tended to experience a greater benefit than those who received platinum-free therapy (*P* = 0.05), and the interaction between HRD status and treatment was significant (*P* = 0.02).

In this study, we also analyzed the correlations of HRD with HRR pathway genes and clinicopathological characteristics. Patients with TNBC with germline *BRCA1/2* mutations showed higher HRD scores than those with somatic *BRCA1/2* mutations (median: 48.0 *vs.* 16.0), and a subsequent analysis indicated that this result might have been due to a higher frequency of LOH in patients with *BRCA1/2* pathogenic germline mutations than in those with somatic variants. Our results also suggested that the contributions of *BRCA1* and *BRCA2* to genomic scarring was distinct, given the significantly higher HRD score observed in g*BRCA1*^mut^ patients than in those carrying g*BRCA2*^mut^ (*P* = 0.02), thus potentially affecting the efficacy of DNA-damaging agents. In 2020, Sokol’s group has reported that *BRCA1* mutations result in more intensive HR deficiency than *BRCA2* mutations^49^. Moreover, a study published in Nature in 2019 has associated *BRCA1* mutations with higher HRD scores than those with *BRCA2* mutations, and associated g*BRCA1* biallelic mutations with higher HRD scores than those with g*BRCA2* biallelic mutations^50^. Although that study did not directly compare the effects of g*BRCA1* and g*BRCA2* mutations on HRD, as was performed herein, their results partially support our finding that g*BRCA1* might be associated with more intensive HR deficiency than g*BRCA2*. Notably, mutations in HRR pathway genes (excluding *BRCA*) had no effect on the HRD scores of patients with TNBC. A high HRD score was significantly correlated with malignant phenotypes, including Ki67 expression, histological grade, and T stage, in agreement with a previous report^51^.

Although both 3D-HRD and the well-known Myriad myChoice HRD assay incorporate LOH, TAI, and LST to evaluate HRD, the 3D-HRD assay might be more cost-effective and applicable and more suitable than the Myriad myChoice HRD assay to evaluate HRD in Chinese patients with cancer. Our study used a well-designed HRD algorithm developed on the basis of sequencing data from Chinese patients with ovarian and breast cancer, with a minimized panel size of 10,000 SNPs. In contrast the Myriad myChoice HRD assay was trained on White patients and included more than 50,000 SNPs^29^. Moreover, the 3D-HRD assay did not include *BRCA1* promoter DNA methylation, which was considered in the Myriad HRD assay. A recent study has found that *BRCA1* promoter DNA methylation is a functionally plastic state that is depleted rapidly after exposure to chemotherapy, and *BRCA1* methylation status was not associated with a survival advantage^52^.

Although our study used a retrospective design, it included 2 clinical cohorts, whose data were obtained in a randomized clinical trial in a highly homogeneous population, which had highly consistent treatment exposures and in a real-world study, thus providing some assurance of data quality and clinical significance. However, prospective studies with larger sample sizes are warranted.

## Conclusions

In HRD-positive patients with TNBC, a platinum-containing regimen may be preferred, and the platinum-free regimen may be more beneficial for HRD negative patients. The HRD status determined according to the HRD score and *BRCA1/2* mutation status has the potential to screen the population suitable for platinum-containing therapy. Notably, this cohort study suggested that HRD may be a more robust predictor of platinum efficacy in the metastatic setting than the adjuvant setting. Further studies with larger cohorts and prospective designs are needed to validate our findings.

## References

[1] Jiang YZ, Liu Y, Xiao Y, Hu X, Jiang L, Zuo WJ (2021). Molecular subtyping and genomic profiling expand precision medicine in refractory metastatic triple-negative breast cancer: the future trial. Cell Res.

[2] Borri F, Granaglia A (2021). Pathology of triple negative breast cancer. Semin Cancer Biol.

[3] Criscitiello C, Azim HA, Schouten PC, Linn SC, Sotiriou C (2012). Understanding the biology of triple-negative breast cancer. Ann Oncol.

[4] Ge J, Zuo W, Chen Y, Shao Z, Yu K (2021). The advance of adjuvant treatment for triple-negative breast cancer. Cancer Biol Med.

[5] Schmid P, Cortes J, Pusztai L, McArthur H, Kummel S, Bergh J (2020). Pembrolizumab for early triple-negative breast cancer. N Engl J Med.

[6] Cortes J, Cescon DW, Rugo HS, Nowecki Z, Im SA, Yusof MM (2020). Pembrolizumab plus chemotherapy versus placebo plus chemotherapy for previously untreated locally recurrent inoperable or metastatic triple-negative breast cancer (KEYNOTE-355): a randomised, placebo-controlled, double-blind, phase 3 clinical trial. Lancet.

[7] Hu N, Si Y, Yue J, Sun T, Wang X, Jia Z (2021). Anlotinib has good efficacy and low toxicity: a phase II study of anlotinib in pre-treated HER-2 negative metastatic breast cancer. Cancer Biol Med.

[8] Zhu A, Yuan P, Hu N, Li M, Wang W, Wang X (2021). Phase II study of apatinib in combination with oral vinorelbine in heavily pretreated HER2-negative metastatic breast cancer and clinical implications of monitoring ctDNA. Cancer Biol Med.

[9] National Comprehensive Cancer Network (2021). NCCN clinical practice guidelines in Oncology. Breast Cancer.

[10] Mayer IA, Zhao F, Arteaga CL, Symmans WF, Park BH, Burnette BL (2021). Randomized phase III postoperative trial of platinum-based chemotherapy versus capecitabine in patients with residual triple-negative breast cancer following neoadjuvant chemotherapy: ECOG-ACRIN EA1131. J Clin Oncol.

[11] Chen Y, Guan Y, Wang J, Ma F, Luo Y, Chen S (2020). Platinum-based chemotherapy in advanced triple-negative breast cancer: a multicenter real-world study in china. Int J Cancer.

[12] Hu X, Wang B, Zhang J, Wang Z, Sun T, Wang S (2020). 282MO Abraxane plus cisplatin compared with gemcitabine plus cisplatin as first-line treatment in patients with metastatic triple-negative breast cancer (GAP): a multicenter, randomized, open-label, phase III trial. Ann Oncol.

[13] Hu XC, Zhang J, Xu BH, Cai L, Ragaz J, Wang ZH (2015). Cisplatin plus gemcitabine versus paclitaxel plus gemcitabine as first-line therapy for metastatic triple-negative breast cancer (CBCSG006): a randomised, open-label, multicentre, phase 3 trial. Lancet Oncol.

[14] Fan Y, Xu BH, Yuan P, Ma F, Wang JY, Ding XY (2013). Docetaxel-cisplatin might be superior to docetaxel-capecitabine in the first-line treatment of metastatic triple-negative breast cancer. Ann Oncol.

[15] Tutt A, Tovey H, Cheang MCU, Kernaghan S, Kilburn L, Gazinska P (2018). Carboplatin in BRCA1/2-mutated and triple-negative breast cancer BRCAness subgroups: the TNT trial. Nat Med.

[16] Li Q, Zhang P, Yuan P, Wang J, Ma F, Luo Y (2015). A phase II study of capecitabine plus cisplatin in metastatic triple-negative breast cancer patients pretreated with anthracyclines and taxanes. Cancer Biol Ther.

[17] O’Shaughnessy J, Schwartzberg L, Danso MA, Miller KD, Rugo HS, Neubauer M (2014). Phase III study of iniparib plus gemcitabine and carboplatin versus gemcitabine and carboplatin in patients with metastatic triple-negative breast cancer. J Clin Oncol.

[18] Zhu Y, Hu Y, Tang C, Guan X, Zhang W (2022). Platinum-based systematic therapy in triple-negative breast cancer. Biochim Biophys Acta Rev Cancer.

[19] Isakoff SJ, Mayer EL, He L, Traina TA, Carey LA, Krag KJ (2015). TBCRC009: a multicenter phase II clinical trial of platinum monotherapy with biomarker assessment in metastatic triple-negative breast cancer. J Clin Oncol.

[20] Byrski T, Gronwald J, Huzarski T, Grzybowska E, Budryk M, Stawicka M (2010). Pathologic complete response rates in young women with BRCA1-positive breast cancers after neoadjuvant chemotherapy. J Clin Oncol.

[21] Arun B, Bayraktar S, Liu DD, Gutierrez Barrera AM, Atchley D, Pusztai L (2011). Response to neoadjuvant systemic therapy for breast cancer in brca mutation carriers and noncarriers: a single-institution experience. J Clin Oncol.

[22] Lord CJ, Ashworth A (2016). BRCAness revisited. Nat Rev Cancer.

[23] Davies H, Glodzik D, Morganella S, Yates LR, Staaf J, Zou X (2017). HRDetect is a predictor of BRCA1 and BRCA2 deficiency based on mutational signatures. Nat Med.

[24] Muggia F, Safra T (2014). ‘BRCAness’ and its implications for platinum action in gynecologic cancer. Anticancer Res.

[25] Tutt AN, Lord CJ, McCabe N, Farmer H, Turner N, Martin NM (2005). Exploiting the DNA repair defect in BRCA mutant cells in the design of new therapeutic strategies for cancer. Cold Spring Harb Symp Quant Biol.

[26] Lord CJ, Ashworth A (2012). The DNA damage response and cancer therapy. Nature.

[27] Taniguchi T, D’Andrea AD (2006). Molecular pathogenesis of fanconi anemia: recent progress. Blood.

[28] Venkitaraman AR (2004). Tracing the network connecting brca and fanconi anaemia proteins. Nat Rev Cancer.

[29] Timms KM, Abkevich V, Hughes E, Neff C, Reid J, Morris B (2014). Association of BRCA1/2 defects with genomic scores predictive of DNA damage repair deficiency among breast cancer subtypes. Breast Cancer Res.

[30] Sipos O, Tovey H, Quist J, Haider S, Nowinski S, Gazinska P (2021). Assessment of structural chromosomal instability phenotypes as biomarkers of carboplatin response in triple negative breast cancer: the TNT trial. Ann Oncol.

[31] Lips EH, Mulder L, Oonk A, van der Kolk LE, Hogervorst FB, Imholz AL (2013). Triple-negative breast cancer: BRCAness and concordance of clinical features with BRCA1-mutation carriers. Br J Cancer.

[32] Popova T, Manie E, Rieunier G, Caux-Moncoutier V, Tirapo C, Dubois T (2012). Ploidy and large-scale genomic instability consistently identify basal-like breast carcinomas with BRCA1/2 inactivation. Cancer Res.

[33] Birkbak NJ, Wang ZC, Kim JY, Eklund AC, Li Q, Tian R (2012). Telomeric allelic imbalance indicates defective DNA repair and sensitivity to DNA-damaging agents. Cancer Discov.

[34] Abkevich V, Timms KM, Hennessy BT, Potter J, Carey MS, Meyer LA (2012). Patterns of genomic loss of heterozygosity predict homologous recombination repair defects in epithelial ovarian cancer. Br J Cancer.

[35] Moore KN, Secord AA, Geller MA, Miller DS, Cloven N, Fleming GF (2019). Niraparib monotherapy for late-line treatment of ovarian cancer (QUADRA): a multicentre, open-label, single-arm, phase 2 trial. Lancet Oncol.

[36] Telli ML, Timms KM, Reid J, Hennessy B, Mills GB, Jensen KC (2016). Homologous recombination deficiency (HRD) score predicts response to platinum-containing neoadjuvant chemotherapy in patients with triple-negative breast cancer. Clin Cancer Res.

[37] Telli ML, Chu C, Badve SS, Vinayak S, Silver DP, Isakoff SJ (2020). Association of tumor-infiltrating lymphocytes with homologous recombination deficiency and BRCA1/2 status in patients with early triple-negative breast cancer: a pooled analysis. Clin Cancer Res.

[38] Loibl S, Weber KE, Timms KM, Elkin EP, Hahnen E, Fasching PA (2018). Survival analysis of carboplatin added to an anthracycline/taxane-based neoadjuvant chemotherapy and HRD score as predictor of response-final results from GeparSixto. Ann Oncol.

[39] Chai Y, Chen Y, Zhang D, Wei Y, Li Z, Li Q (2022). Homologous recombination deficiency (HRD) and BRCA 1/2 gene mutation for predicting the effect of platinum-based neoadjuvant chemotherapy of early-stage triple-negative breast cancer (TNBC): a systematic review and meta-analysis. J Pers Med.

[40] Mayer EL, Abramson V, Jankowitz R, Falkson C, Marcom PK, Traina T (2020). TBCRC 030: a phase II study of preoperative cisplatin versus paclitaxel in triple-negative breast cancer: evaluating the homologous recombination deficiency (HRD) biomarker. Ann Oncol.

[41] Zhang L, Chen Y, Cheng MY, Zhuang X, Zou J, Wei D (2022). Homologous recombination deficiency predicts the response to platinum-based neoadjuvant chemotherapy in early-stage triple-negative breast cancer patients: a systematic review and meta-analysis. Ther Adv Med Oncol.

[42] Ueno T, Kitano S, Masuda N, Ikarashi D, Yamashita M, Chiba T (2022). Immune microenvironment, homologous recombination deficiency, and therapeutic response to neoadjuvant chemotherapy in triple-negative breast cancer: Japan Breast Cancer Research Group (JBCRG)22 TR. BMC Med.

[43] Vetter M, Fokas S, Biskup E, Schmid T, Schwab F, Schoetzau A (2017). Efficacy of adjuvant chemotherapy with carboplatin for early triple negative breast cancer: a single center experience. Oncotarget.

[44] Yu KD, Ye FG, He M, Fan L, Ma D, Mo M (2020). Effect of adjuvant paclitaxel and carboplatin on survival in women with triple-negative breast cancer: a phase 3 randomized clinical trial. JAMA Oncol.

[45] Zhao EY, Shen Y, Pleasance E, Kasaian K, Leelakumari S, Jones M (2017). Homologous recombination deficiency and platinum-based therapy outcomes in advanced breast cancer. Clin Cancer Res.

[46] Du F, Wang W, Wang Y, Li M, Zhu A, Wang J (2020). Carboplatin plus taxanes are non-inferior to epirubicin plus cyclophosphamide followed by taxanes as adjuvant chemotherapy for early triple-negative breast cancer. Breast Cancer Res Treat.

[47] Hong R, Ma F, Xu B, Li Q, Zhang P, Yuan P (2014). Efficacy of platinum-based chemotherapy in triple-negative breast cancer patients with metastases confined to the lungs: a single-institute experience. Anticancer Drugs.

[48] Lheureux S, Lai Z, Dougherty BA, Runswick S, Hodgson DR, Timms KM (2017). Long-term responders on olaparib maintenance in high-grade serous ovarian cancer: clinical and molecular characterization. Clin Cancer Res.

[49] Sokol ES, Pavlick D, Khiabanian H, Frampton GM, Ross JS, Gregg JP (2020). Pan-cancer analysis of BRCA1 and BRCA2 genomic alterations and their association with genomic instability as measured by genome-wide loss of heterozygosity. JCO Precis Oncol.

[50] Priestley P, Baber J, Lolkema MP, Steeghs N, de Bruijn E, Shale C (2019). Pan-cancer whole-genome analyses of metastatic solid tumours. Nature.

[51] Knijnenburg TA, Wang L, Zimmermann MT, Chambwe N, Gao GF, Cherniack AD (2018). Genomic and molecular landscape of DNA damage repair deficiency across The Cancer Genome Atlas. Cell Rep.

[52] Menghi F, Banda K, Kumar P, Straub R, Dobrolecki L, Rodriguez IV (2022). Genomic and epigenomic BRCA alterations predict adaptive resistance and response to platinum-based therapy in patients with triple-negative breast and ovarian carcinomas. Sci Transl Med.

