# Supplementary material for: Association between homologous recombination deficiency and outcomes with platinum and platinum-free chemotherapy in patients with triple-negative breast cancer
Source: Cancer Biol Med. 2023 Mar 2;20(2):155–68. doi: 10.20892/j.issn.2095-3941.2022.0525 (PMC9978893; doi:10.20892/j.issn.2095-3941.2022.0525)
Supplement: Supplementary file 1 [file cbm-20-155-s001.docx]

**Supplementary materials**

[METHODS 2](#_Toc92730017)

[Figure S1. A Flow Chart Illustrating the Study Design 5](#_Toc92730018)

[Figure S2. Distribution of HRD Score in the Combined Ovarian (n=488) and Breast (n=106) Cancer Training Set 6](#_Toc92730019)

[Figure S3. Repeatability and Reproducibility of HRD-panel assay 7](#_Toc92730020)

[Figure S4. Correlation between HRD Score and Clinicopathological Features in the Combined Surgical and Metastatic TNBC Cohort (n=189) 8](#_Toc92730021)

[Figure S5. Correlation between HRD Score and Clinicopathological Features in the Combined Surgical and Metastatic TNBC Cohort (n=189) 9](#_Toc92730022)

[Figure S6. Lollipop Plot Showing Mutations in *BRCA1/2* Detected from the Combined Surgical and Metastatic TNBC Cohort (n=189) 10](#_Toc92730023)

[Figure S7. The Association of HRD Score with *BRCA1/2* and LOH 11](#_Toc92730024)

[Figure S8. Scatter Plot of HRD Scores in TNBC Cohort with *BRCA1/2* Status. 12](#_Toc92730025)

[Figure S9. Frequency of HRR-Related Gene Mutations in the Combined Surgical and Metastatic TNBC Cohort (n=189). 13](#_Toc92730026)

[Figure S10. Association of HRD Score with HRR-Relate Genes. 14](#_Toc92730027)

[Figure S11. Association between *PIK3CA* Mutation and Clinical Outcomes in TNBC Patients Treated with Platinum-Containing Chemotherapy (Surgical Cohort n=149). 15](#_Toc92730028)

[Figure S12. Association of HRD Status with Objective Response Rate (ORR) and Disease Control Rate (DCR) upon First-Line Chemotherapy in Metastatic TNBC 16](#_Toc92730029)

[Figure S13. Kaplan–Meier Estimates Overall Survival (OS) of Metastatic TNBC 17](#_Toc92730030)

[Figure S14. Disease-Free Survival (DFS) of *BRCA1/2*^wt^ TNBC Receiving Adjuvant Chemotherapy in the Surgical Cohort 18](#_Toc92730031)

[Table S1. The Gene List of 733 Panel. 19](#_Toc92730032)

[Table S2. Pathogenic/Likely Pathogenic *BRCA1/2* Mutations in the Combined Surgical and Metastatic TNBC cohort (n=189). 20](#_Toc92730033)

[Table S3. The Gene List of 15 HRR-Relate Genes. 21](#_Toc92730034)

[Table S4. Frequency of Pathogenic/Likely Pathogenic Alterations in HRD+ and HRD- Tumor in the combined TNBC cohort (n=189). 22](#_Toc92730035)

[Table S5. Baseline Demographics and Disease Characteristics (metastatic cohort). 23](#_Toc92730036)

[Table S6. Progression-Free Survival analysis based on HRD status (metastatic cohort). 24](#_Toc92730037)

[Table S7. Univariable Analysis of Progression-Free Survival in Platinum-Containing group (metastasis cohort). 25](#_Toc92730038)

[Table S8. Baseline Demographics and Disease Characteristics (surgical cohort). 26](#_Toc92730039)

[Table S9. Disease-Free Survival analysis based on HRD status and BRCA status (surgical cohort) 27](#_Toc92730040)

[Table S10. Univariable of Disease-Free Survival in the Platinum-Containing Group (Surgical Cohort). 28](#_Toc92730041)

# METHODS

***Next generation sequencing and sequencing data analysis***

DNA preparation, sequencing, and data analysis were conducted at 3D Medicines, Inc., a College of American Pathologists (CAP)-accredited and Clinical Laboratory Improvement Amendments (CLIA)-certified laboratory. Briefly, DNA was extracted from formalin-fixed paraffin-embedded (FFPE) tissue samples using the ReliaPrep™ FFPE gDNA Miniprep System (Promega). DNA extracts (30-200 ng) were sheared to 250 bp fragments and libraries were constructed with xGenPrism DNA Library Prep Kit (Integrated DNA Technologies, Inc.). Indexed libraries were then subjected to probe-based hybridization with a customized NGS panel targeting 733 cancer-related genes (3D Medicines). The captured libraries were loaded onto a NovaSeq 6000 platform (Illumina) for 100 bp paired-end sequencing. Burrows-Wheeler Aligner (v0.7.12) was employed to align raw data to reference human genome hg19. PCR duplicate reads were removed and sequence metrics were collected using Picard (v1.130) and SAMtools (v1.1.19), respectively. Somatic single nucleotide variants (SNVs) were detected using an in-house developed R package to execute a variant detection model based on binomial test. Local realignment was performed to detect indels. Variants were then filtered by their unique supporting read depth, strand bias, and base quality as previously described[^1^](#_ENREF_1). All variants were then filtered using an automated false positive filtering pipeline to ensure sensitivity and specificity at an allele frequency (AF) of ≥1%. Single-nucleotide polymorphism (SNPs) and indels were annotated by ANNOVAR using databases of dbSNP (v138), 1000Genome, and ESP6500 (population frequency > 0.015). Copy number variations (CNVs) and gene rearrangements were detected as described previously [^1^](#_ENREF_1). All insertions, deletions, and SNVs in the coding region of targeted genes, including in-frame and frameshift mutations, missense, silent, stop loss, stopgain and splice in the target intron were considered. Pathogenic or likely pathogenic mutation was determined as per a joint consensus of the latest literature or reports from clinical trials and the recommendation of American College of Medical Genetics and Genomics and the Association for Molecular Pathology (ACMG-AMP)[^2^](#_ENREF_2). Germline variants were identified using paired normal tissues adjacent to the cancer.

***HRD Score Calculation***

HR deficiency (HRD) score was determined using three genomic instability markers, including loss of heterozygosity (LOH), telomeric-allelic imbalance (TAI), and large-scale state transitions (LST).

HRD score = TAI score+ LST score +LOH score

***TAI, LST, and LOH Calculation***

TAI, LST, and LOH were calculated in reference to previous literature[^3^](#_ENREF_3). Algorithm modifications were made from the initial reporting of TAI and LST calculations. TAI (number of regions of allelic imbalance that extend to one of the subtelomeres) and LST (number of breakpoints between regions longer than 10 Mb) scores were calculated using the allelic imbalance profiles. LOH was calculated according to the number of subchromosomal LOH regions longer than 15 Mb.

***HRD cutoff***

The threshold score of HRD was determined at 30 to identify 95% of patients with known *BRAC1/2* deficiency in the training cohort. We used 594 clinical tissue samples with known *BRCA1/2* mutation status to be detected using the targeted cancer panel spanning over 10,000 SNP.

***HRD status***

A tumor was defined as HRD-positive if having had an HRD score of ≥30 and/or deleterious mutation in *BRCA1/2*, while a *BRCA1/2*-intact tumor with an HRD score of < 30 was defined as HRD-negative.

***Limit of detection of the 3D-HRD assay***

Cell lines HCC1954 and HC1143 were purchased from ATCC (http://www.atcc.org/). Two cell line dilution pools were made by their matched normal cell lines. The tumor purity gradients were 40%, 30%, 20%, 10%, and 5%. When the tumor purity was ≥ 20%, HRD-positive status was called out from both of the two cell lines (500X mean coverage). Therefore, the LOD was determined to be 20% tumor purity.

***Repeatability and reproducibility***

The repeatability and reproducibility of the assay were evaluated using three HRD-positive clinical tissue samples and three HRD-negative clinical tissue samples. For each sample, two batches of test were conducted by two groups, with three duplicates in each batch.

|  | Sample ID | HRD score |
| --- | --- | --- |
| HRD-Pos | Sample 1 | 87 |
|  | Sample 2 | 61 |
|  | Sample 3 | 40 |
| HRD-Neg | Sample 4 | 18 |
|  | Sample 5 | 12 |
|  | Sample 6 | 1 |

***Treatment efficacy evaluation***

Response was assessed as per the Response Evaluation Criteria in Solid Tumors (RECIST), version 1.1, and categorized as complete response (CR), partial response (PR), stable disease (SD), and disease progression (PD). Objective response rate (ORR) was defined as the proportion of patients who achieved a complete or partial response as their best overall response. Patients with unknown response status were considered nonresponders. Disease control rate (DCR) was defined as the proportion of patients who achieved a stable disease or complete response or partial response. Progression-free survival (PFS) was defined as the time from the onset of chemotherapy to disease progression or death, whichever occurred first. Disease-free survival (DFS) was defined as the time from the onset of chemotherapy to disease recurrence or death, whichever occurred first. Overall survival (OS) was defined as the time from the onset of chemotherapy to death as a result of any cause.

***PD-L1, Ki67 expression by Immunohistochemistry***

The method was similar to those published in our previous articles[^4-6^](#_ENREF_4). All samples taken from surgery were fixed with formalin and embedded in paraffin. Four-um-thick sections were cut from a paraffin block of each specimen and attached to slides for H&E and IHC. The sections were de-paraffinized, rehydrated, and rinsed in distilled water, and then treated with 3% hydrogen peroxide solution for 10 min. Antigen retrieval was achieved by heating in a water bath with EDTA. PD-L1 staining procedure was performed using the Dako Autostainer Link 48 platform and an automated staining protocol validated for the PD-L1 IHC 22C3 pharmDx assay (Dako North America)^7^. Positivity was defined as complete circumferential or partial cell membrane staining in ≥1% tumor cells and/or tumor-associated immune cells. Immunohistochemical staining of Ki67 (using Ki67 antibody diluted 1:200; Beijing Zhongshan Golden Bridge Biotechnology Co., Ltd., Beijing, China), Ki67 staining was analyzed under optical microscopy (Olympus, Barrington, NJ, USA).

**Reference**

1. Su D, Zhang D, Chen K, et al. High performance of targeted next generation sequencing on variance detection in clinical tumor specimens in comparison with current conventional methods. *Journal of experimental & clinical cancer research : CR.* 2017;36(1):121.

2. Richards S, Aziz N, Bale S, et al. Standards and guidelines for the interpretation of sequence variants: a joint consensus recommendation of the American College of Medical Genetics and Genomics and the Association for Molecular Pathology. *Genetics in medicine : official journal of the American College of Medical Genetics.* 2015;17(5):405-424.

3. Timms KM, Abkevich V, Hughes E, et al. Association of *BRCA1/2* defects with genomic scores predictive of DNA damage repair deficiency among breast cancer subtypes. *Breast cancer research : BCR.* 2014;16(6):475.

4. Chen Y, Guan Y, Wang J, et al. Platinum-based chemotherapy in advanced triple-negative breast cancer: A multicenter real-world study in China. *International journal of cancer.* 2020;147(12):3490-3499.

5. Du F, Wang W, Wang Y, et al. Carboplatin plus taxanes are non-inferior to epirubicin plus cyclophosphamide followed by taxanes as adjuvant chemotherapy for early triple-negative breast cancer. *Breast cancer research and treatment.* 2020;182(1):67-77.

6. Yuan P, Xu B, Wang C, Zhang C, Sun M, Yuan L. Ki-67 expression in luminal type breast cancer and its association with the clinicopathology of the cancer. *Oncology letters.* 2016;11(3):2101-2105.

7. Hayes DF, Thor AD, Dressler LG, et al. HER2 and response to paclitaxel in node-positive breast cancer. *The New England journal of medicine.* 2007;357(15):1496-1506.

# Figure S1. A Flow Chart Illustrating the Study Design

A flow chart showing the study design and samples used in each step. Of the 40 patients in the metastatic cohort, 21 and 19 patients received platinum-containing and platinum-free chemotherapy, respectively, in the first-line advanced setting. Of the 149 patients in the surgical cohort, 74 were treated with platinum-containing regimen and 75 with platinum-free chemotherapy as adjuvant treatment.

Predictive value of HRD status for therapeutic efficacy (Platinum-containing and platinum-free therapy)

Association of HRD with genomic signatures and clinicopathological features

LOD;

Sensitivity and specificity;

Repeatability and reproducibility

HRD definition, cutoff training

Tissue samples from 594 breast cancer and ovarian cancer patients with known BRCA1/2 mutation status

Algorithm development

Metastatic cohort（N=40)

Surgical cohort (N=149)

Metastatic cohort（N=40)

Surgical cohort (N=149)

Technical validation

Cell line HCC1954 and HCC1143;

75 tumor samples from breast and ovarian cancer patients with known BRCA1/2 mutation status

Therapeutic outcome relevance

HRD prevalence

# Figure S2. Distribution of HRD Score in the Combined Ovarian (n=488) and Breast (n=106) Cancer Training Set

The threshold score of HRD was determined to 30 to identify 95% of patients with known *BRAC1/2*-deficient mutations in the combined training cohort. *BRCA*-deficient was defined as pathogenic or likely pathogenic *BRCA1/2* mutations, with LOH in the wild-type copy. Orange color denotes *BRCA1/2*-deficient; grey color denotes *BRCA1/2*-intact. HRD, homologous recombination deficiency; LOH, loss of heterozygosity; TAI, telomeric allelic imbalance; LST, large-scale state transition.

**
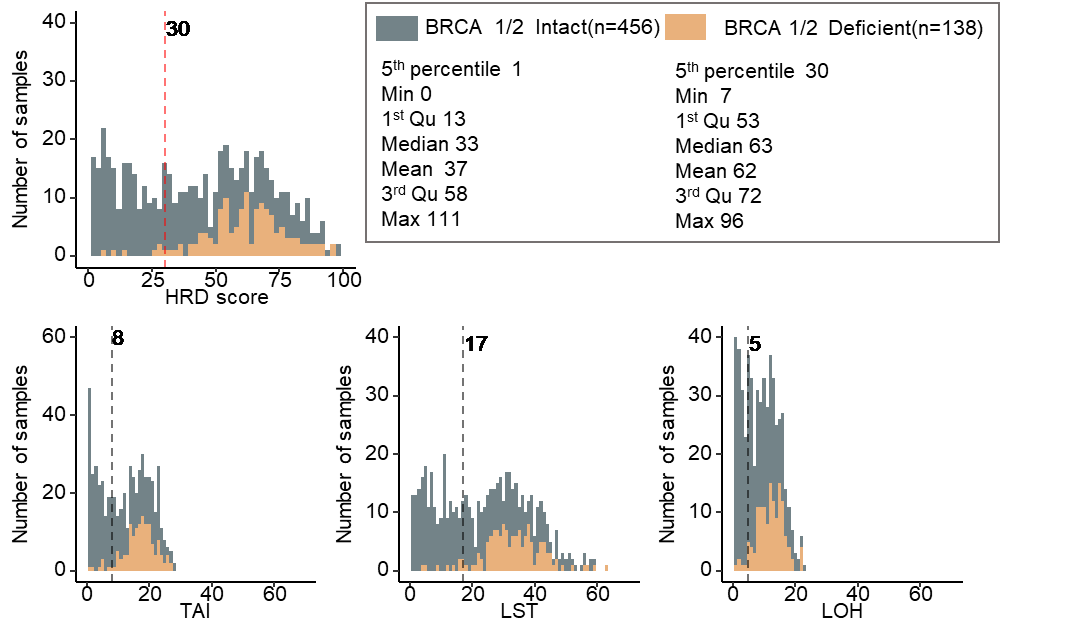
**

# Figure S3. Repeatability and Reproducibility of HRD-panel assay

6 tumor samples with known *BRCA1/2* status were subjected to HRD-panel testing and identified as HRD-positive or HRD-negative according to the threshold of 30 determined in the training set. For each sample, two batches of test were conducted by two groups, with three duplicates in each batch. Blue line, HRD cutoff-value 30; red line, mean HRD score of the six tests; red dots, HRD score of the three replicates in the first batch; green dots, HRD score of the three replicates in the second batch.


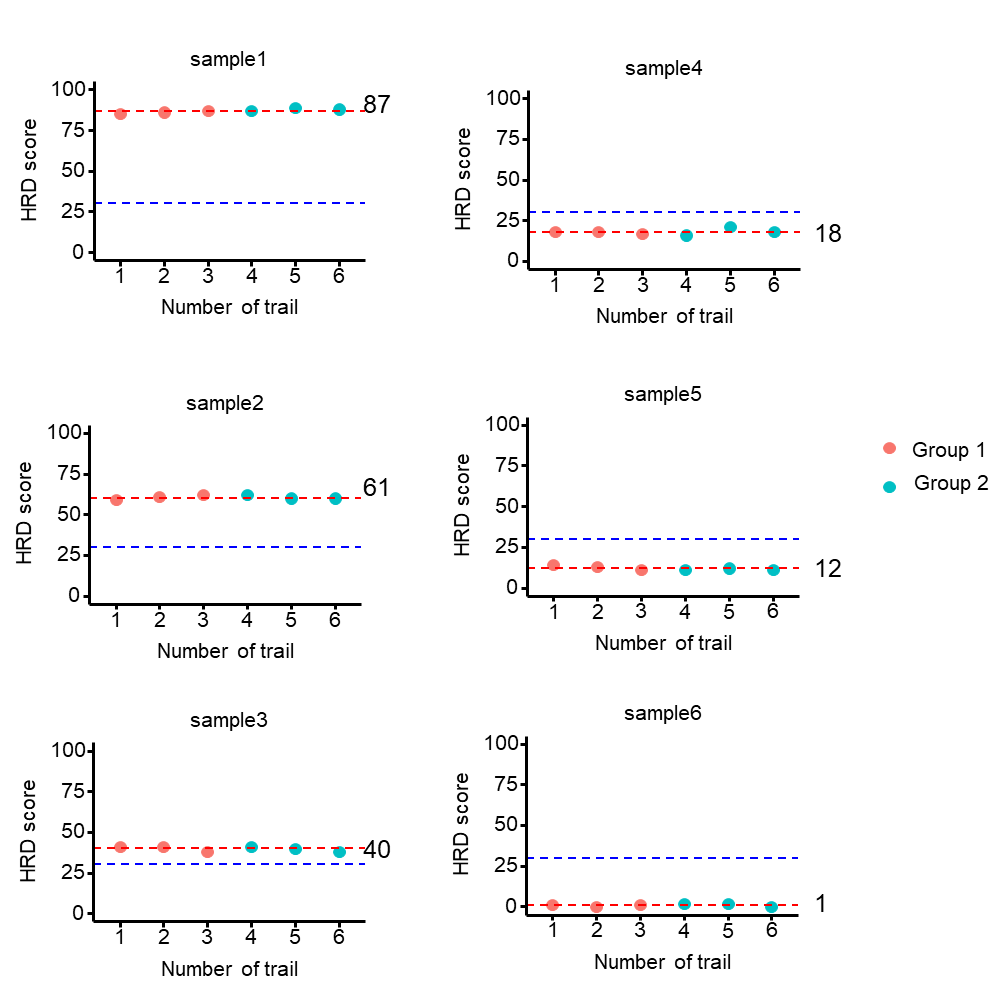


# Figure S4. Correlation between HRD Score and Clinicopathological Features in the Combined Surgical and Metastatic TNBC Cohort (n=189)

A, Association between HRD-score and Ki67 expression index. B−D, Association between HRD score and histological grade (B), tumor TNM stage (C), and T stage (D). HRD score was determined by 3D-HRD assay. Differences in groups were calculated by wilcox test. Spearman regression was performed to analyze the relationship between HRD score and Ki67 proliferation index (%). *P* < 0.05 was considered statistically significant.


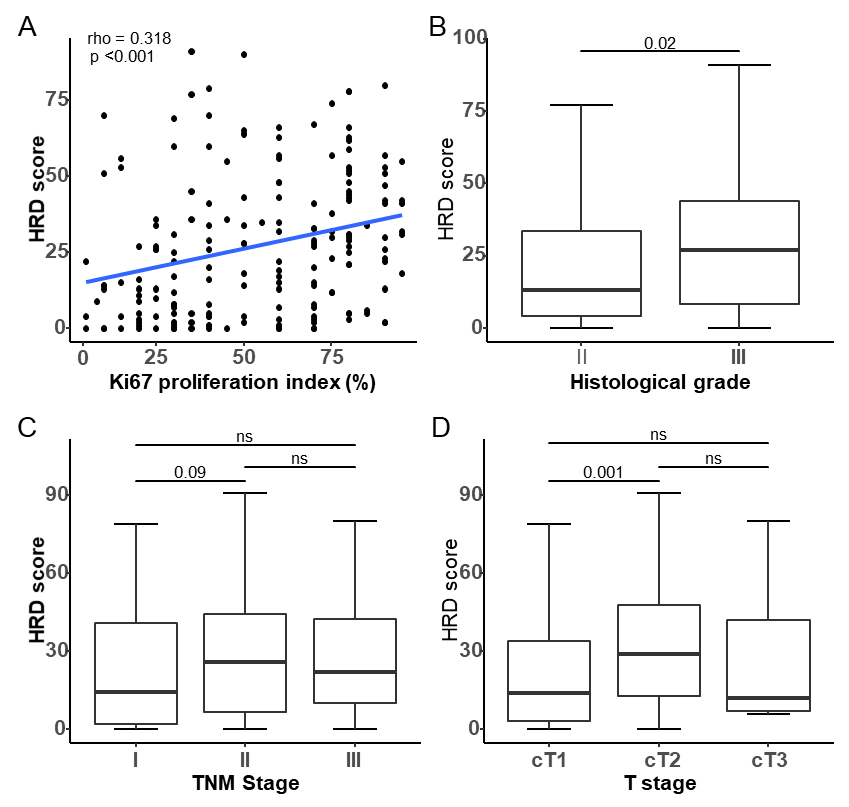


# Figure S5. Correlation between HRD Score and Clinicopathological Features in the Combined Surgical and Metastatic TNBC Cohort (n=189)

A−G, Association of HRD score with age (A), Ki67 expression index (B), N stage (C), lymph node metastasis status (D), menopausal status (E), PD-L1 TPS (F), and PD-L1 IPS (G). PD-L1, programmed death ligand 1; TPS, tumor proportion score, the proportion of viable tumor cells showing partial or complete membrane PD-L1 staining at any intensity; IPS, immune proportion score (IPS), the percent of tumor-infiltrating immune cells with PD-L1 staining at any intensity. Differences in groups were calculated and presented either by Wilcox test or t-tests, as appropriate. *P* < 0.05 was considered statistically significant.

.
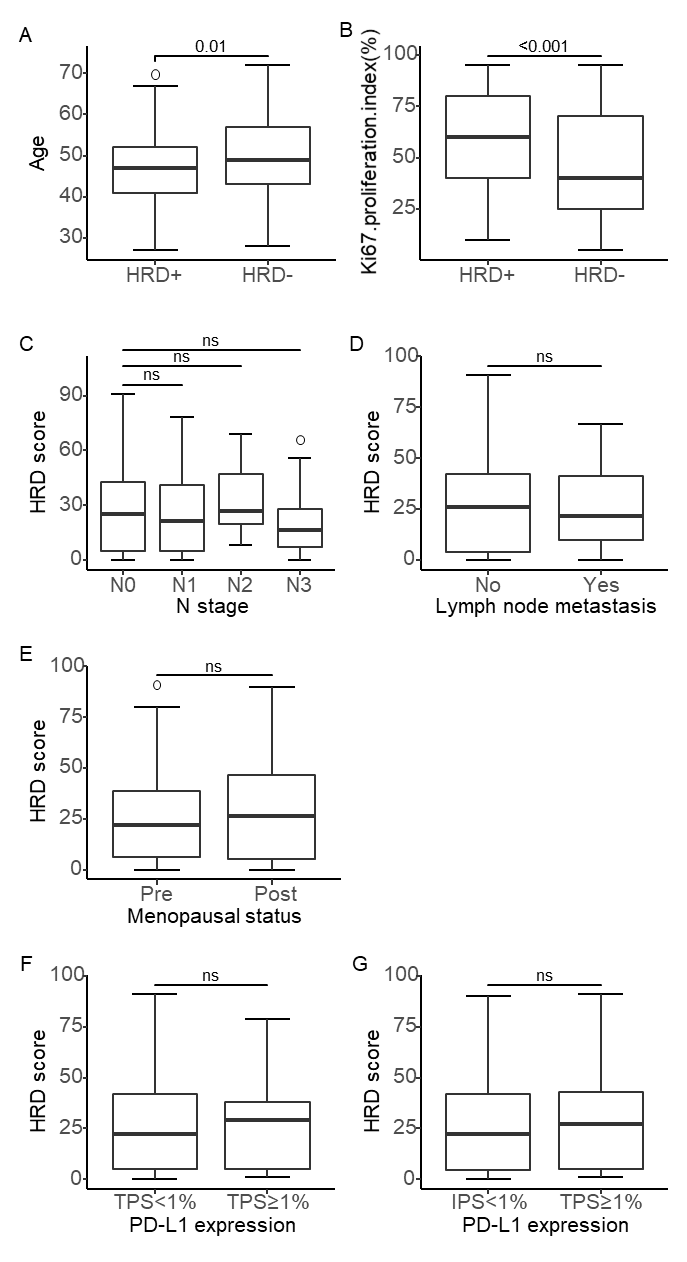


# Figure S6. Lollipop Plot Showing Mutations in *BRCA1/2* Detected from the Combined Surgical and Metastatic TNBC Cohort (n=189).


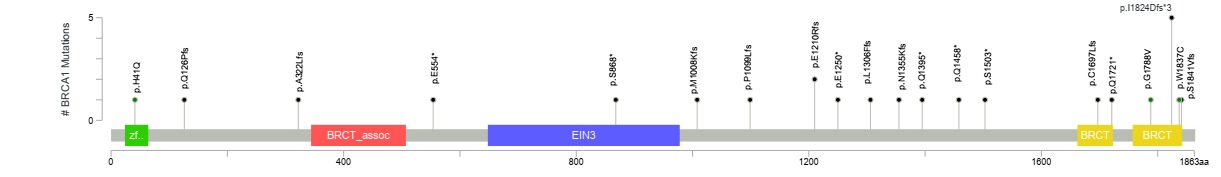

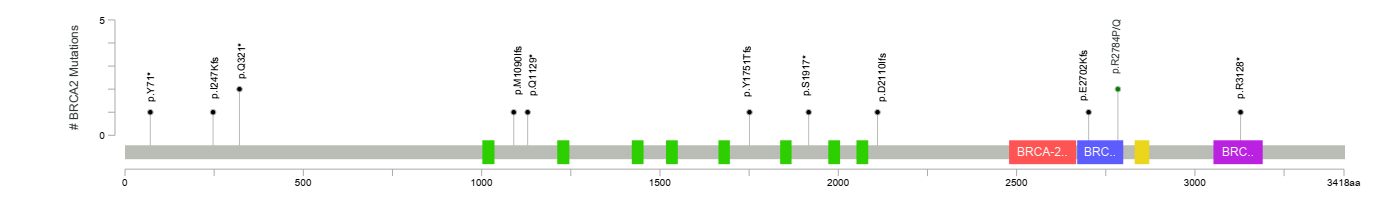


# Figure S7. The Association of HRD Score with *BRCA1/2* and LOH

The correlation of HRD score with *BRCA1/2* and LOH was analyzed using the combined resectable and metastatic TNBC cohort (n=189). A, HRD score of *BRCA*^wt^ and *BRCA*^mut^ tumors. B, HRD score of tumors with germline and somatic mutations in *BRCA1* and *BRCA2*. C, Frequency of LOH in tumors harboring *BRCA1* and *BRCA2* mutations. D, HRD score of *BRCA1* or *BRCA2*-mutated tumors with different LOH status. LOH, loss of heterozygosity. Differences in groups were calculated and presented either by Wilcox test or chi-squared test, as appropriate. *P* < 0.05 was considered statistically significant.


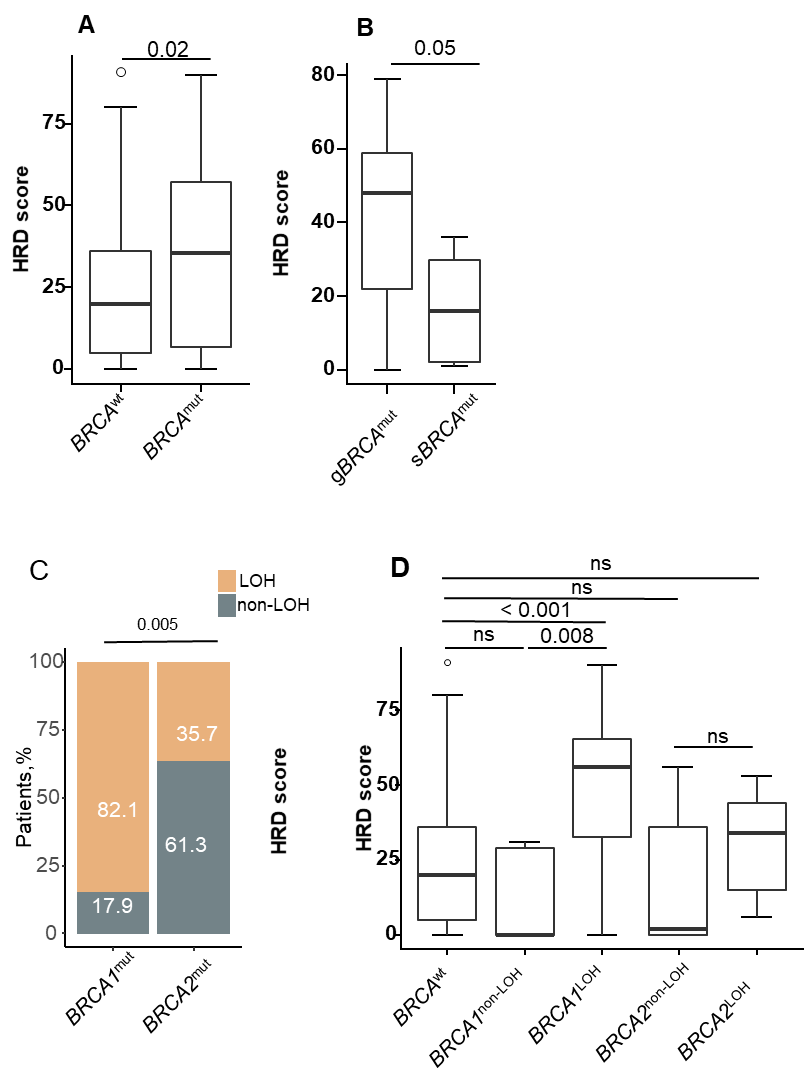


Figure S8. Scatter Plot of HRD Scores in TNBC Cohort with *BRCA1/2* Status.

Dots are colored according to *BRCA1/2* status. Red, germline *BRCA1* mutant; orange, somatic *BRCA1* mutant; blue, germline *BRCA2* mutant; purple, somatic *BRCA2* mutant; green, with two *BRCA1/2* mutations; gray, *BRCA1/2* wild-type.

**
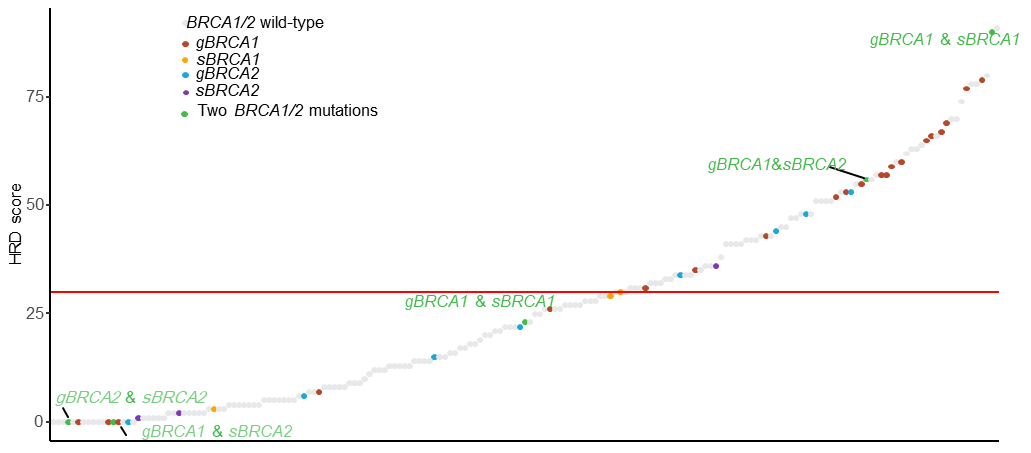
**

# Figure S9. Frequency of HRR-Related Gene Mutations in the Combined Surgical and Metastatic TNBC Cohort (n=189).

A, Overall mutation rate of HRR germline and somatic mutations. Distribution of HRR-related germline mutations (B) and somatic mutations (C) in TNBC patients (n=189). HRR, homologous recombination repair. Different color represents diverse mutation gene and percentage.


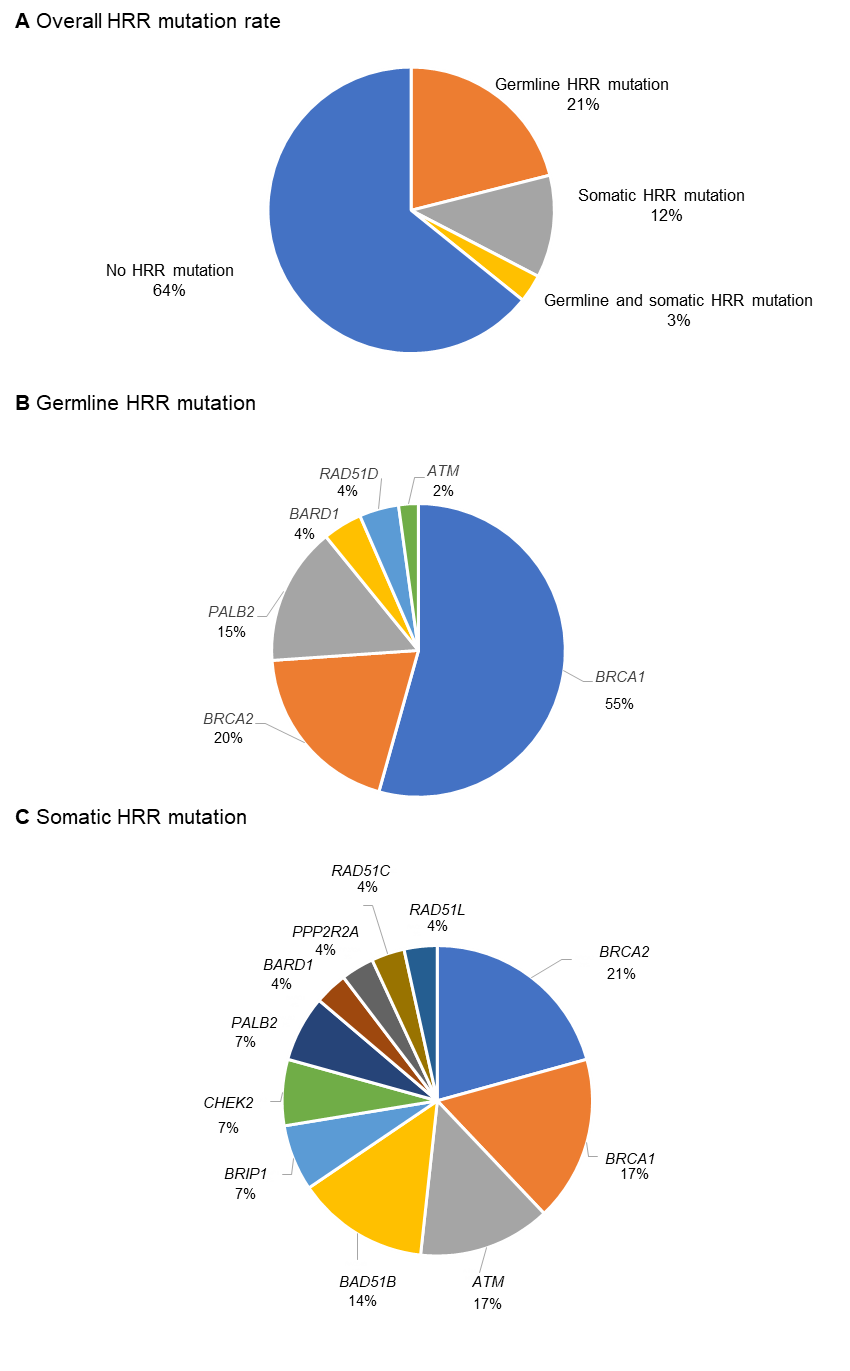


# Figure S10. Association of HRD Score with HRR-Relate Genes.

The correlation of HRD score with HRR-related gene mutations was analyzed using the combined surgical and metastatic TNBC cohort (n=189). A, HRD score of tumors with mutations in HRR-related genes and the wild-type counterpart tumors. B, HRD score of tumor with HRR-related gene mutations excluding *BRCA*. C, Scatter plots showing the HRD score of tumors with HRR-relate gene mutations. HRR, homologous recombination repair. Dots color represents the HRR-gene mutation type as indicated. Green, *BRCA1/2* mutant; red, *PALB2* mutant; blue, *RAD51* gene family; orange, *ATM* mutant; purple, other HRR gene mutants; gray, HRR gene wild-type. Differences in groups were calculated and presented by wilcox test. P < 0.05 was considered statistically significant.


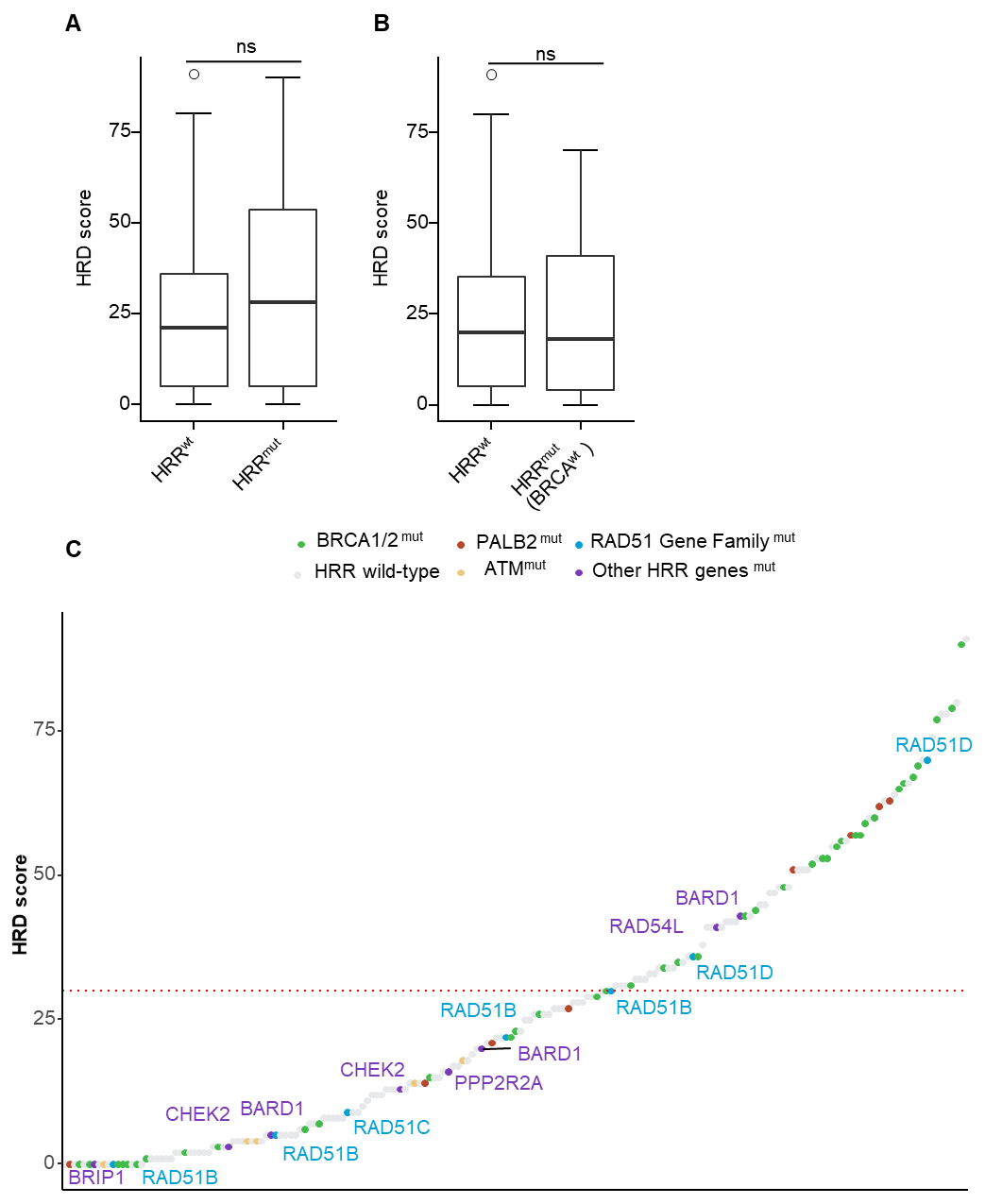


# Figure S11. Association between *PIK3CA* Mutation and Clinical Outcomes in TNBC Patients Treated with Platinum-Containing Chemotherapy (Surgical Cohort n=149).

A, Disease-free survival of *PIK3CA*^mut^ and *PIK3CA*^wt^ patients receiving platinum-containing adjuvant chemotherapy. B, Disease-free survival of *PIK3CA*^mut^ and *PIK3CA*^wt^ patients receiving platinum-free adjuvant chemotherapy.


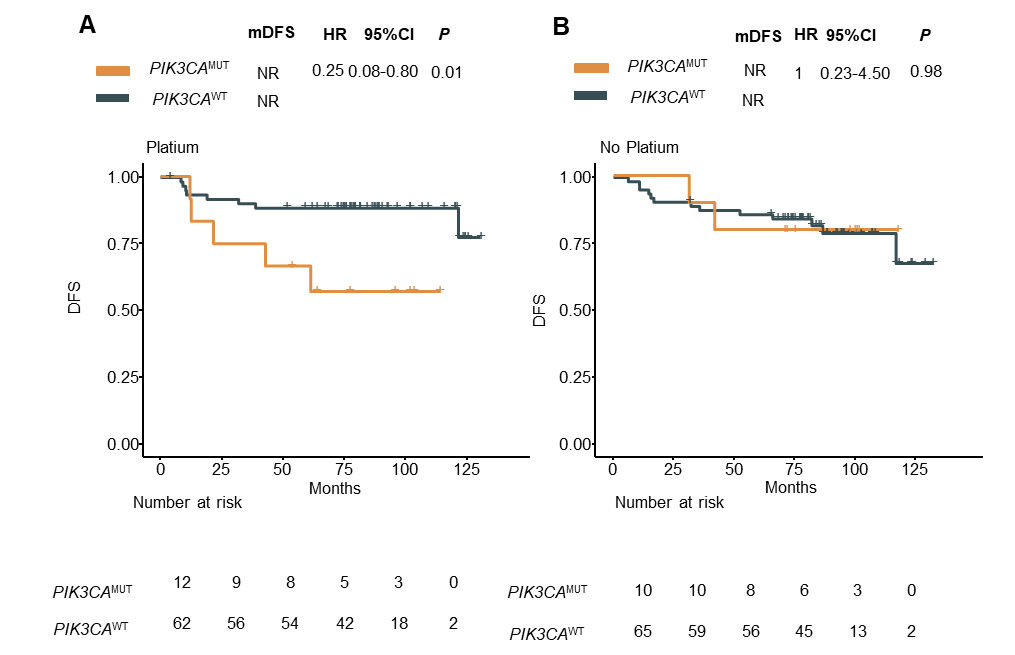


# Figure S12. Association of HRD Status with Objective Response Rate (ORR) and Disease Control Rate (DCR) upon First-Line Chemotherapy in Metastatic TNBC

HRD+, defined as having a HRD score of ≥30 and/or deleterious mutation in *BRCA1/2*, while a *BRCA1/2*-intact tumor with an HRD score of < 30 was defined as HRD-.

**
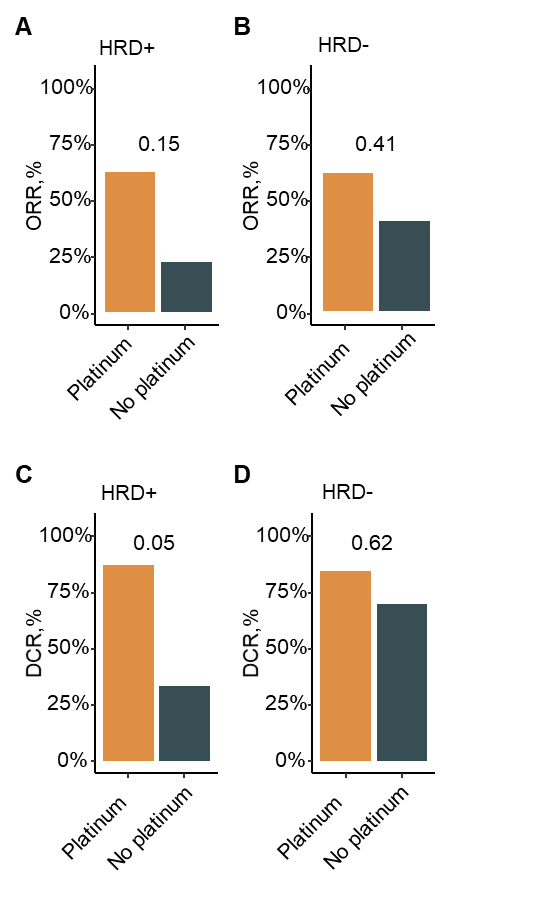
**

# Figure S13. Kaplan–Meier Estimates Overall Survival (OS) of Metastatic TNBC

A, OS of patients receiving first-line platinum-based regimen in comparison to platinum-free chemotherapy. B-C, OS of HRD+ (B) and HRD- (C) patients receiving platinum-containing and platinum-free chemotherapy. HRD+, defined as having a HRD score of ≥30 and/or deleterious mutation in *BRCA1/2*, while a *BRCA1/2*-intact tumor with an HRD score of < 30 was defined as HRD-.


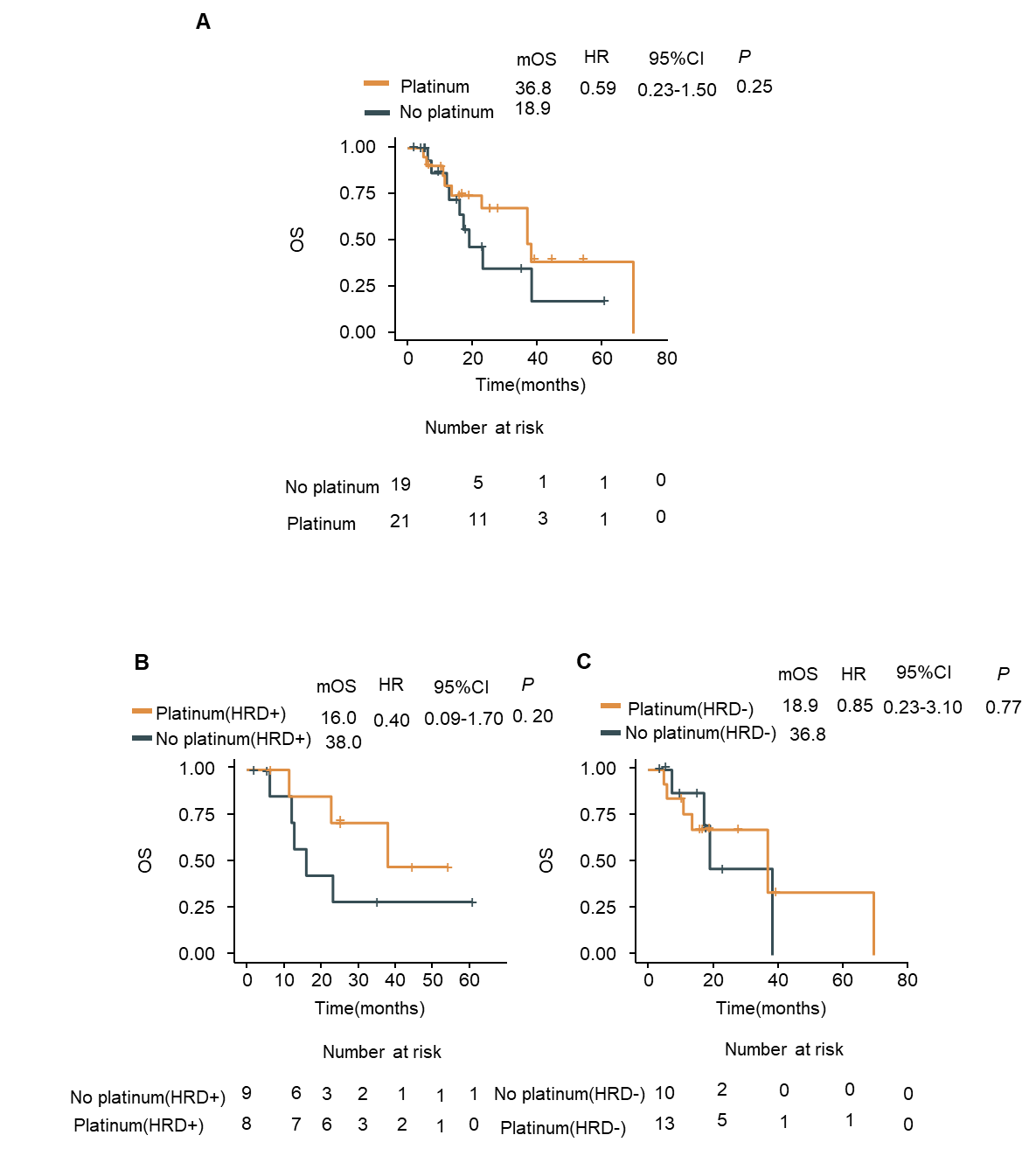


# Figure S14. Disease-Free Survival (DFS) of *BRCA1/2*^wt^ TNBC Receiving Adjuvant Chemotherapy in the Surgical Cohort

HRD+ was defined as having a HRD score of ≥30 and/or deleterious mutation in *BRCA1/2*, while a *BRCA1/2*-intact tumor with an HRD score of < 30 was defined as HRD-.


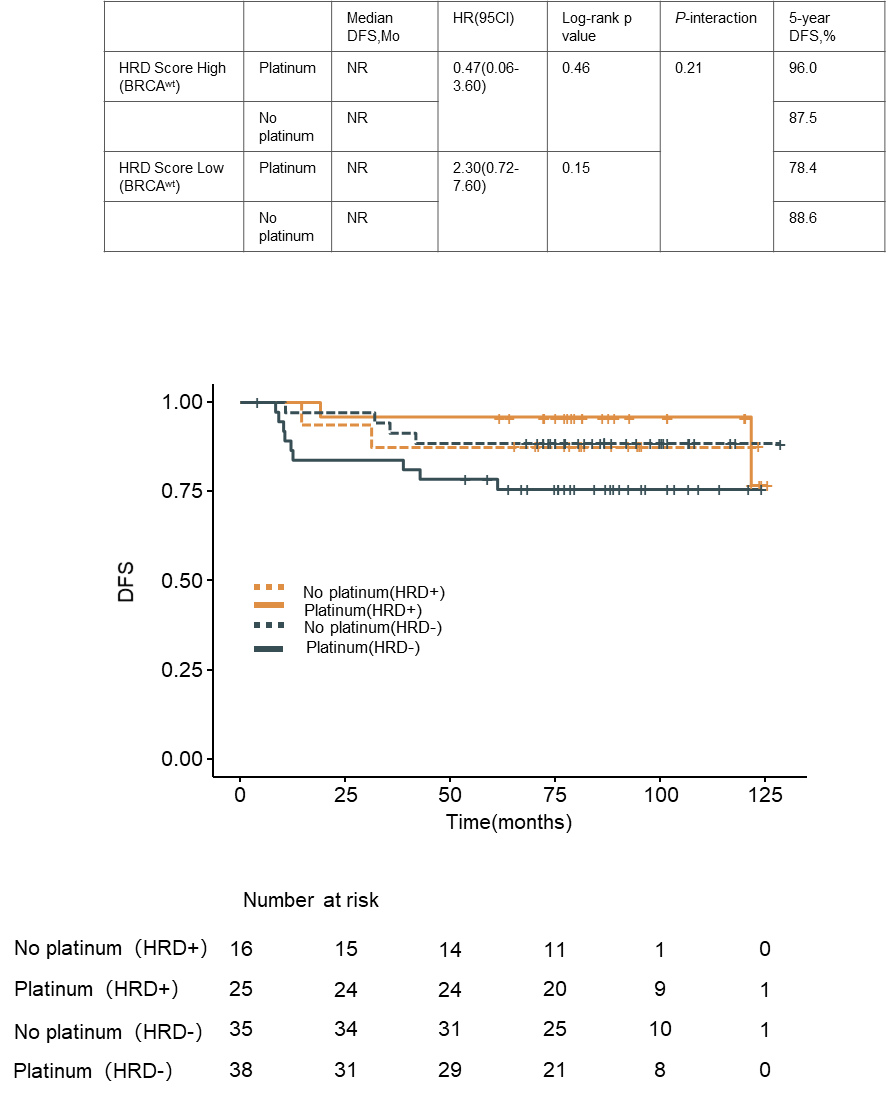


# Table S1. The Gene List of 733 Panel.

| **Gene list** |
| --- |
| *ABCB11 ABI1 ABL1 ABRAXAS1 ACKR3 ACSL3 ACVR1 ACVR1B ACVR2A AEN AFF3 AFF4 AKT1 AKT2 AKT3 ALK ALKBH2 ALKBH3 AMER1 ANK1 APC APEX1 APEX2 APLF APOBEC3B APTX AR ARAF AREG ARHGAP5 ARID1A ARID1B ARID2 ARNT ASXL1 ATM ATP1A1 ATP2B3 ATR ATRIP ATRX AURKA AXIN1 AXIN2 AXL B2M BAP1 BARD1 BAZ1A BCL10 BCL11A BCL11B BCL2 BCL2L1 BCL2L11 BCL6 BCOR BCORL1 BCR BIRC3 BIRC5 BLM BMP5 BMPR1A BRAF BRCA1 BRCA2 BRD4 BRIP1 BTG1 BTK BUB1B CACNA1D CALR CAMTA1 CANT1 CARD11 CARS CASP8 CBFA2T3 CBFB CBL CBLB CCDC6 CCNB1IP1 CCND1 CCND2 CCND3 CCNE1 CCNH CCNO CD274 CD74 CD79A CD79B CDC73 CDH1 CDH10 CDH11 CDK12 CDK2 CDK4 CDK6 CDK7 CDK8 CDKN1A CDKN1B CDKN1C CDKN2A CDKN2B CDKN2C CDX2 CEBPA CENPS CENPX CETN2 CHAF1A CHD1 CHD2 CHD4 CHEK1 CHEK2 CHIC2 CIC CIITA CLIP1 CLK2 CLTCL1 CNBP CNOT3 COL7A1 CRBN CREB3L1 CREB3L2 CREBBP CRKL CRLF2 CRNKL1 CRTC1 CRTC3 CSF1R CSF3R CTCF CTNNB1 CTNND2 CTR9 CUL1 CUL3 CUL4A CUL5 CUX1 CXCR4 CYLD CYP17A1 CYP2C19 CYP2D6 CYSLTR2 DAXX DCLRE1A DCLRE1B DCLRE1C DDB1 DDB2 DDIT3 DDR2 DDX10 DDX3X DDX5 DDX6 DICER1 DIS3 DIS3L2 DKC1 DMC1 DNM2 DNMT1 DNMT3A DNTT DOCK8 DPYD DROSHA DUT EBF1 EED EGFR EIF3E EIF4A2 ELANE ELF3 ELF4 ELK4 ELL ELOA EME1 EME2 EMSY ENDOV EP300 EPAS1 EPCAM EPHA2 EPHA3 EPHA7 EPHB1 EPS15 ERBB2 ERBB3 ERBB4 ERC1 ERCC1 ERCC2 ERCC3 ERCC4 ERCC5 ERCC6 ERCC8 EREG ERF ERRFI1 ESR1 ETNK1 ETV1 ETV4 ETV5 ETV6 EWSR1 EXO1 EXT1 EXT2 EZH2 EZR FAAP100 FAAP20 FAAP24 FAH FAM135B FAM47C FAN1 FANCA FANCB FANCC FANCD2 FANCE FANCF FANCG FANCI FANCL FANCM FAS FAT1 FAT4 FBXW7 FEN1 FES FGF19 FGF3 FGF4 FGFR1 FGFR2 FGFR3 FGFR4 FH FHIT FLCN FLT1 FLT3 FLT4 FOXA1 FOXL2 FOXP1 FRK FRS2 FUBP1 FUS G6PD GALNT12 GAS7 GATA1 GATA2 GATA3 GBA GEN1 GFI1 GJB2 GLI1 GLI2 GLI3 GNA11 GNA13 GNAQ GNAS GPC3 GRB2 GREM1 GRIN2A GSK3B GSTT1 GTF2H1 GTF2H3 GTF2H4 GTF2H5 H2AFX H3F3A HDAC1 HDAC2 HELQ HES1 HEY1 HFE HFM1 HGF HIF1A HIP1 HIST1H3B HLTF HMBS HMGA2 HMGB1 HNF1A HNRNPA2B1 HOOK3 HOXA11 HOXB13 HRAS HUS1 HUS1B IDH1 IDH2 IGF1R IGF2 IKBKE IKZF1 IL6ST IL7R INPP4B IRS2 ITGAV ITK JAK1 JAK2 JAK3 JMJD1C JUN KCNJ5 KDM5A KDM5C KDM6A KDR KEAP1 KIT KLF4 KMT2A KMT2C KMT2D KNL1 KRAS LASP1 LATS1 LATS2 LCK LEF1 LIFR LIG1 LIG3 LIG4 LMNA LMO1 LRP1B LZTR1 MAD2L2 MAP2K1 MAP2K2 MAP2K4 MAP3K1 MAPK1 MAX MBD4 MCL1 MDC1 MDM2 MDM4 MECOM MED12 MEF2B MEN1 MET MGA MGMT MITF MLH1 MLH3 MLLT3 MLST8 MMS19 MNAT1 MPG MPL MPLKIP MRE11 MSH2 MSH3 MSH4 MSH5 MSH6 MTAP MTOR MUS81 MUTYH MYB MYC MYCL MYCN MYD88 MYOD1 NAB2 NABP2 NBN NCOA3 NCOR1 NCOR2 NDRG1 NEIL1 NEIL2 NEIL3 NF1 NF2 NFE2L2 NFIB NFKBIA NHEJ1 NHP2 NKX2-1 NME1 NONO NOP10 NOTCH1 NOTCH2 NOTCH3 NOTCH4 NPM1 NR4A3 NRAS NRG1 NRG3 NSD2 NSD3 NT5C2 NTHL1 NTRK1 NTRK2 NTRK3 NUDT1 NUP93 NUTM1 OGG1 PAK1 PALB2 PARP1 PARP2 PARP3 PARP4 PAX3 PAX5 PAX7 PAX8 PBRM1 PCDH9 PCNA PDCD1LG2 PDGFB PDGFRA PDGFRB PDPK1 PER1 PER2 PER3 PHF6 PHOX2B PICALM PIK3CA PIK3CB PIK3CD PIK3R1 PIK3R2 PIK3R3 PIM1 PLCG2 PLXNA1 PLXNB1 PML PMS1 PMS2 PNKP POLB POLD1 POLD3 POLD4 POLE POLE2 POLE3 POLE4 POLG POLH POLI POLK POLL POLM POLN POLQ POT1 POU2AF1 POU5F1 PPARG PPM1D PPP2R1A PPP2R2A PPP4R1 PPP4R2 PPP4R3A PPP4R3B PPP4R4 PPP6C PRCC PRDM1 PRDM16 PRDM9 PREX2 PRF1 PRKACA PRKAR1A PRKCH PRKDC PRPF19 PRSS1 PSIP1 PTCH1 PTEN PTK2 PTK6 PTPN11 PTPN13 PTPRD PTPRT QKI RAC1 RAD1 RAD18 RAD21 RAD23A RAD23B RAD50 RAD51 RAD51B RAD51C RAD51D RAD52 RAD54B RAD54L RAD54L2 RAD9A RAD9B RAF1 RANBP2 RAP1GDS1 RARA RASA1 RB1 RBBP8 RBM10 RBX1 RDM1 RECQL RECQL4 RECQL5 RET REV1 REV3L RFC1 RFC2 RFC3 RFC4 RFC5 RFWD3 RGS7 RHBDF2 RHEB RHOA RHOH RICTOR RIF1 RIT1 RMI1 RMI2 RNF168 RNF213 RNF4 RNF43 RNF8 ROS1 RPA1 RPA2 RPA3 RPA4 RPS6KA3 RPS6KB1 RPTOR RRM2B RUNX1 RUNX1T1 RXRA SBDS SDC4 SDHA SDHAF2 SDHB SDHC SDHD SEM1 SERPINA1 SERPINB3 SETBP1 SETD2 SETMAR SF3B1 SFPQ SGK1 SH2B3 SH2D1A SHOC2 SHPRH SLC25A13 SLC29A1 SLC34A2 SLC45A3 SLIT2 SLX1A SLX4 SMAD2 SMAD3 SMAD4 SMARCA1 SMARCA2 SMARCA4 SMARCB1 SMO SMUG1 SOCS1 SOS1 SOX2 SOX9 SPEN SPO11 SPOP SPRED1 SPRTN SPTA1 SRC SRGAP3 SRSF2 SRY SS18 STAG2 STAT3 STK11 SUFU SUZ12 SYK TBL1XR1 TBX3 TCF3 TCF7L2 TCL1A TDG TDP1 TDP2 TEAD2 TELO2 TERT TET1 TET2 TFE3 TGFBR1 TGFBR2 THBS2 TIMELESS TMEM127 TMEM189 TMPRSS2 TNFAIP3 TOP2A TOP3A TOP3B TOPBP1 TP53 TP53BP1 TP63 TPMT TRAF7 TREX1 TREX2 TRIM37 TSC1 TSC2 TSHR TSPAN31 TYK2 U2AF1 UBE2A UBE2B UBE2N UBE2T UBE2V2 UGT1A1 UNG UROD USP1 USP6 USP8 UVSSA VEGFA VHL WAS WDR48 WIF1 WRN WT1 XAB2 XPA XPC XPO1 XRCC1 XRCC2 XRCC3 XRCC4 XRCC5 XRCC6 YAP1 YWHAE ZBTB16 ZFHX3 ZNF217 ZNF479 ZNF703 ZNF750 ZNRF3* |

# Table S2. Pathogenic/Likely Pathogenic *BRCA1/2* Mutations in the Combined Surgical and Metastatic TNBC cohort (n=189).

| **Patient No.** | **Gene** | **Alterations** |
| --- | --- | --- |
| 1 | *BRCA2* | c.8104-8141del |
| 2 | *BRCA1* | c.212+1G>T |
| 3 | *BRCA1* | c.5193+1G>T |
| 4 | *BRCA2* | c.3385C>T |
| 5 | *BRCA1* | c.5521del |
| 5 | *BRCA2* | c.740-767del |
| 6 | *BRCA1* | c.1660G>T |
| 7 | *BRCA2* | c.213T>G |
| 8 | *BRCA2* | c.5250del |
| 9 | *BRCA2* | c.3270del |
| 9 | *BRCA2* | c.6327del |
| 10 | *BRCA1* | c.5511G>C |
| 11 | *BRCA1* | c.3294del |
| 12 | *BRCA1* | Loss of copy number |
| 13 | *BRCA1* | c.5470-5477del |
| 14 | *BRCA1* | c.374dup |
| 15 | *BRCA1* | c.123C>A |
| 16 | *BRCA1* | c.3627dup |
| 17 | *BRCA2* | c.8351G>A |
| 18 | *BRCA1* | c.4065-4068del |
| 19 | *BRCA1* | c.5161C>T |
| 20 | *BRCA1* | c.3748G>T |
| 21 | *BRCA1* | c.5090del |
| 22 | *BRCA2* | c.961C>T |
| 23 | *BRCA1* | c.5470-5477del |
| 24 | *BRCA2* | c.961C>T |
| 25 | *BRCA1* | c.964del |
| 25 | *BRCA1* | c.3021-3058del |
| 26 | *BRCA1* | c.3917dup |
| 27 | *BRCA1* | c.5470-5477del |
| 28 | *BRCA1* | c.5470-5477del |
| 29 | *BRCA2* | c.8351G>C |
| 30 | *BRCA1* | c.5470-5477del |
| 30 | *BRCA1* | Loss of copy number |
| 31 | *BRCA2* | c.8331+1G>A |
| 32 | *BRCA1* | c.5363G>T |
| 33 | *BRCA1* | c.3627dup |
| 34 | *BRCA1* | c.4183C>T |
| 35 | *BRCA1* | c.4372C>T |
| 36 | *BRCA1* | c.5193+1G>T |
| 37 | *BRCA2* | c.9382C>T |
| 38 | *BRCA2* | c.5750C>G |
| 39 | *BRCA1* | c.2603C>G |
| 40 | *BRCA2* | Loss of copy number |
| 40 | *BRCA1* | c.4508C>A |

# Table S3. The Gene List of 15 HRR-Relate Genes.

| **Classification** | **15 Genes** |
| --- | --- |
| **Homologous**  **recombination**  **Repair (HRR)** | *ATM, BARD1, BRCA1, BRCA2, BRIP1, CDK12, CHEK1, CHEK2, FANCL, PALB2, PPP2R2A, RAD51B, RAD51D, RAD51C, RAD54L* |

# Table S4. Frequency of Pathogenic/Likely Pathogenic Alterations in HRD+ and HRD- Tumor in the combined TNBC cohort (n=189).

| Gene | Alterations | Mutated in  HRD+ (n) | Mutated in  HRD- (n) | Wild-type in HRD+ (n) | Wild-type in  HRD- (n) | P-value |
| --- | --- | --- | --- | --- | --- | --- |
| *TP53* | SNV, Indel | 83 | 76 | 10 | 20 | 0.07 |
| *MYC* | CNG | 18 | 15 | 75 | 81 | 0.57 |
| *BRCA1* | SNV, Indel, CNL | 28 | 0 | 65 | 96 | <0.001 |
| *PIK3CA* | SNV, Indel, CNG | 5 | 22 | 88 | 74 | 0.001 |
| *MCL1* | CNG | 10 | 7 | 83 | 89 | 0.45 |
| *FANCC* | SNV, Indel | 5 | 10 | 88 | 86 | 0.28 |
| *BRCA2* | SNV, Indel, CNL | 14 | 0 | 79 | 96 | <0.001 |
| *PTEN* | SNV, Indel, CNL | 7 | 6 | 86 | 90 | 0.78 |
| *RB1* | SNV, Indel | 7 | 5 | 86 | 91 | 0.56 |
| *CXCR4* | CNG | 3 | 8 | 90 | 88 | 0.21 |
| *NF1* | SNV, Indel | 3 | 8 | 90 | 88 | 0.21 |
| *PIK3R1* | SNV, Indel, CNL | 5 | 6 | 88 | 90 | 1.00 |
| *RIT1* | CNG | 6 | 5 | 87 | 91 | 0.77 |
| *EGFR* | SNV, Indel, CNG, Fusion | 6 | 4 | 87 | 92 | 0.53 |
| *FAM135B* | CNG | 5 | 5 | 88 | 91 | 1.00 |
| *CHD2* | SNV, Indel | 3 | 6 | 90 | 90 | 0.50 |
| *KMT2C* | SNV, Indel | 3 | 6 | 90 | 90 | 0.50 |
| *PREX2* | CNG | 4 | 5 | 89 | 91 | 1.00 |
| *PTK2* | CNG | 4 | 5 | 89 | 91 | 1.00 |
| *ATRX* | SNV, Indel | 1 | 7 | 92 | 89 | 0.06 |
| *CDKN2A* | SNV, Indel, CNL | 2 | 6 | 91 | 90 | 0.28 |
| *PALB2* | SNV, Indel | 4 | 4 | 89 | 92 | 1.00 |
| *FGFR1* | CNG | 2 | 5 | 91 | 91 | 0.45 |
| *LRP1B* | SNV, Indel | 4 | 3 | 89 | 93 | 0.72 |
| *ATR* | SNV, Indel | 2 | 4 | 91 | 92 | 0.68 |
| *CHD4* | SNV, Indel | 3 | 3 | 90 | 93 | 1.00 |
| *DDR2* | CNG | 5 | 1 | 88 | 95 | 0.11 |
| *NCOR2* | Indel | 3 | 3 | 90 | 93 | 1.00 |
| *AKT1* | SNV, CNG | 1 | 4 | 92 | 92 | 0.37 |
| *ATM* | SNV, Indel | 0 | 5 | 93 | 91 | 0.06 |
| *BIRC5* | CNG | 5 | 0 | 88 | 96 | 0.03 |
| *CCND2* | CNG | 2 | 3 | 91 | 93 | 1.00 |
| *FGFR2* | SNV, Gain, Fusion | 2 | 3 | 91 | 93 | 1.00 |
| *KRAS* | CNG | 1 | 4 | 92 | 92 | 0.37 |
| *NCOR1* | SNV, Indel | 0 | 5 | 93 | 91 | 0.06 |
| *AKT3* | CNG | 2 | 2 | 91 | 94 | 1.00 |
| *ARID2* | Indel | 1 | 3 | 92 | 93 | 0.62 |
| *BRAF* | SNV, CNG | 2 | 2 | 91 | 94 | 1.00 |
| *CCNE1* | CNG | 1 | 3 | 92 | 93 | 0.62 |
| *CDKN2B* | CNL | 2 | 2 | 91 | 94 | 1.00 |
| *CREBBP* | SNV, Indel, CNL | 3 | 1 | 90 | 95 | 0.36 |
| *JAK2* | SNV, Indel, CNG | 2 | 2 | 91 | 94 | 1.00 |
| *KDM5A* | CNG | 2 | 2 | 91 | 94 | 1.00 |
| *PDCD1LG2* | CNG | 4 | 0 | 89 | 96 | 0.06 |
| *RAD51* | SNV | 0 | 4 | 93 | 92 | 0.12 |
| *RAD51B* | SNV, Indel | 1 | 3 | 92 | 93 | 0.62 |
| *RECQL4* | SNV, Indel | 2 | 2 | 91 | 94 | 1.00 |
| *ROS1* | CNG, Fusion | 2 | 2 | 91 | 94 | 1.00 |
| *SOX2* | CNG | 4 | 0 | 89 | 96 | 0.06 |
| *ZNF703* | CNG | 2 | 2 | 91 | 94 | 1.00 |

Abbreviations: HRD+, defined as having a HRD score of ≥30 and/or deleterious mutation in BRCA1/2, while a BRCA1/2-intact tumor with an HRD score of < 30 was defined as HRD-; Homologous recombination deficiency positive status; SNV, single nucleotide variation; Indel, insertion or deletion of nucleotides; CNG, copy number gain; CNL, copy number loss.

# Table S5. Baseline Demographics and Disease Characteristics (metastatic cohort).

|  | | HRD+ | | | | HRD- | | | |
| --- | --- | --- | --- | --- | --- | --- | --- | --- | --- |
| Characteristics | | Platinum (n =8) | | No platinum (n=9) | | Platinum (n =13) | | No platinum (n =10) | |
| **Age, mean (SD), year** | | 42.3 (6.2) | | 47.3 (9.9) | | 46.4 (7.5) | | 48.8 (9.1) | |
| **Ki67 proliferation index (%)** | |  | |  | |  | |  | |
| Median(range) | | 50 (10, 85) | | 80 (15, 90) | | 60 (5, 80) | | 32 (20, 95) | |
| N/A | | 0 | | 0 | | 1 | | 0 | |
| **ECOG score** | |  | |  | |  | |  | |
| 0 | | 5 (62.5) | | 4 (44.4) | | 9 (69.2) | | 8 (80.0) | |
| I | | 3 (37.5) | | 4 (44.4) | | 3 (23.1) | | 2 (20.0) | |
| II | | 0 (0) | | 1 (11.1) | | 1 (7.7) | | 0 (0) | |
| **Menopausal status** | |  | |  | |  | |  | |
| Postmenopausal | | 3 (37.5) | | 4 (44.4) | | 8 (61.5) | | 6 (60.0) | |
| Premenopausal | | 5 (62.5) | | 5 (55.6) | | 5 (38.5) | | 4 (40.0) | |
| **Prior chemotherapy in the neoadjuvant setting** | |  | |  | |  | |  | |
| Yes | | 0 (37.5) | | 4 (44.4) | | 2 (15.4) | | 2 (20.0) | |
| Containing platinum | | 0 | | 1 | | 0 | | 2 | |
| No | | 8 (100) | | 5 (55.6) | | 11 (84.6) | | 8 (80.0) | |
| **Prior chemotherapy in the adjuvant setting** | |  | |  | |  | |  | |
| Yes | | 8(100) | | 5 (55.6) | | 11 (84.6) | | 10 (100) | |
| Containing platinum | | 0 | | 1 | | 1 | | 2 | |
| No | | 0 | | 4 (44.4) | | 2 (15.4) | | 0 (0) | |
| **Postoperative radiotherapy** | |  | |  | |  | |  | |
| Yes | | 2 (25.0) | | 5 (55.6) | | 9 (69.2) | | 6 (60.0) | |
| No | | 6 (75.0) | | 4 (44.4) | | 4 (30.8) | | 4 (40.0) | |
| **HRD score** | |  | |  | |  | |  | |
| Low (<30) | | 1 (12.5) | | 0 (0) | | 13 (100) | | 10 (100) | |
| High (≥30) | | 7 (87.5) | | 9 (100) | | 0 (0) | | 0 (0) | |
| **BRCA1/2** | |  | |  | |  | |  | |
| Mutant | | 3 (37.5) | | 2 (22.2) | | 0 (0) | | 0 (0) | |
| Wild-type | | 5 (62.5) | | 7 (77.8) | | 13 (100) | | 10 (100) | |
| **HRR-related genes** | |  | |  | |  | |  | |
| Mutant | | 3 (37.5) | | 2 (22.2) | | 1 (7.7) | | 1 (10.0) | |
| Wild-type | | 5 (62.5) | | 7 (77.8) | | 12 (92.3) | | 9 (90.0) | |

Abbreviations: ECOG score, Eastern Cooperative Oncology Group performance score; HRD, homologous recombination deficiency; HRR, homologous recombination repair; N/A, not available. A tumor was defined as HRD+ if having a HRD score of ≥30 and/or deleterious mutation in BRCA1/2, while a BRCA1/2-intact tumor with an HRD score of < 30 was defined as HRD-.

# Table S6. Progression-Free Survival analysis based on HRD status (metastatic cohort).

**A PFS stratified by HRD status and treatment**

|  |  | Median PFS, Month | HR(95%CI) | Log-rank *p* value |
| --- | --- | --- | --- | --- |
| HRD+ | Platinum | 13.6 | 0.11(0.02-0.51) | 0.001 |
|  | No platinum | 2.0 |  |  |
| HRD- | Platinum | 6.8 | 0.88(0.70-2.10) | 0.77 |
|  | No platinum | 4.5 |  |  |

**B PFS of patients with BRCA wild-type tumors**

|  |  | Median PFS, Month | HR(95%CI) | Log-rank  *p* value |
| --- | --- | --- | --- | --- |
| HRD Score High  (BRCA^wt^) | Platinum | 14.2 | 0.10(0.01-0.84) | 0.01 |
|  | No platinum | 2.2 |  |  |
| HRD Score Low  (BRCA^wt^) | Platinum | 6.8 | 0.88(0.37-2.10) | 0.77 |
|  | No platinum | 4.5 |  |  |

Abbreviations: HRD, homologous recombination deficiency; PFS, Progression-Free Survival. A tumor was defined as HRD+ if having a HRD score of ≥30 and/or deleterious mutation in BRCA1/2, while a BRCA1/2-intact tumor with an HRD score of < 30 was defined as HRD-.

# Table S7. Univariable Analysis of Progression-Free Survival in Platinum-Containing group (metastasis cohort).

| Characteristics | Level | Univariable analysis | | |  |
| --- | --- | --- | --- | --- | --- |
|  |  | Hazard ratio | | 95% CI | *P* value |
| HRD status | HRD-  (n=13) |  | Ref |  | 0.06 |
|  | HRD+  (n=8) | 0.36 | | 0.12-1.03 |  |
| Age, year | <50  (n=16) | Ref | |  | 0.39 |
|  | ≥50  (n=5) | 1.58 | | 0.56-4.50 |  |
| BRCA 1/2 | Wild-type  (n=18) | Ref | |  | 0.57 |
|  | Mutant  (n=3) | 0.70 | | 0.20-2.44 |  |
| HRR-related gens | Wild-type  (n=17) | Ref | |  | 0.74 |
|  | Mutant  (n=4) | 0.83 | | 0.27-2.52 |  |
| ECOG score | 0  (n=14) | Ref | |  | 0.23 |
|  | I-II  (n=7) | 1.78 | | 0.70-4.58 |  |
| Ki67 | <30  (n=4) | Ref | |  | 0.64 |
|  | ≥30  (n=16) | 1.35 | | 0.38-4.76 |  |

Abbreviations: HRD, homologous recombination deficiency; HRR, homologous recombination repair; ECOG score, Eastern Cooperative Oncology Group performance score. A tumor was defined as HRD+ if having a HRD score of ≥30 and/or deleterious mutation in BRCA1/2, while a BRCA1/2-intact tumor with an HRD score of < 30 was defined as HRD-.

# Table S8. Baseline Demographics and Disease Characteristics (surgical cohort).

|  | HRD+ | | HRD- | |
| --- | --- | --- | --- | --- |
| Characteristics | Platinum  （n=36）  (n =36) | No platinum (n =40) | Platinum (n =38) | No platinum (n =35) |
| **Age, mean (SD), year** | 48.9 (10.4) | 45.3 (7.3) | 50.4 (9.6) | 51.5 (10.7) |
| **Ki67 proliferation index (%)** |  |  |  |  |
| Median(range) | 75.0 (10, 95) | 60.0(10, 95) | 40.0(5, 90) | 40.0(5, 90) |
| N/A | 1 | 1 | 1 | 1 |
| **Pathological status** |  |  |  |  |
| Ductal | 34 (94.4) | 38 (95.0) | 38 (100) | 34 (97.1) |
| Medullary | 1 (2.8) | 1 (2.5) | 0 (0) | 1 (2.9) |
| Others | 1 (2.8) | 1 (2.5) | 0 (0) | 0 (0) |
| **Histological Grade** |  |  |  |  |
| II | 5 (14.3) | 8 (20.0) | 10 (26.3) | 15 (44.1) |
| III | 30 (85.7) | 32 (80.0) | 28 (73.7) | 19 (55.9) |
| N/A | 1 | 0 | 0 | 1 |
| **Intravascular cancer embolus** |  |  |  |  |
| No | 32 (88.9) | 28 (77.8) | 35 (92.1) | 29 (85.3) |
| Yes | 4 (11.1) | 8 (22.2) | 3 (7.9) | 5 (14.7) |
| N/A | 0 | 4 | 0 | 1 |
| **Stage** |  |  |  |  |
| I | 14 (38.9) | 8 (20.0) | 14 (36.8) | 14 (40.0) |
| II | 22 (61.1) | 30 (75.0) | 21 (55.3) | 18 (51.4) |
| III | 0 (0) | 2 (5.0) | 3 (7.9) | 3 (8.6) |
| **Menopausal status** |  |  |  |  |
| Postmenopausal | 17 (48.6) | 12 (30.8) | 17 (43.6) | 20 (55.6) |
| Premenopausal | 19 (51.4) | 28 (69.2) | 21 (56.4) | 15 (44.4) |
| **HRD score** |  |  |  |  |
| Low (<30) | 5 (13.9) | 10 (25.0) | 38 (100) | 35 (100) |
| High (≥30) | 31 (86.1) | 30 (75.0) | 0 (0) | 0 (0) |
| **BRCA1/2** |  |  |  |  |
| Mutant | 11 (30.6) | 24 (60.0) | 0 (0) | 0 (0) |
| Wild-type | 25 (69.4) | 16 (40.0) | 38(100) | 35 (100) |
| **HRR-related genes** |  |  |  |  |
| Mutant | 17 (47.2) | 27 (67.5) | 13 (34.2) | 4 (11.4) |
| Wild-type | 19 (52.8) | 13 (32.5) | 25(65.8) | 31 (88.6) |

Abbreviations: A tumor was defined as HRD+ if having a HRD score of ≥30 and/or deleterious mutation in BRCA1/2, while a BRCA1/2-intact tumor with an HRD score of < 30 was defined as HRD-; HRR, Homologous recombination repair. N/A, not available

# Table S9. Disease-Free Survival analysis based on HRD status and BRCA status (surgical cohort)

**A DFS stratified by HRD status and treatment**

|  |  | Median DFS, Month | HR(95%CI) | Log-rank *p* value | *P*-interaction | 5-year DFS,% |
| --- | --- | --- | --- | --- | --- | --- |
| HRD+ | Platinum | NR | 0.33(0.11-1.10) | 0.05 | 0.02 | 91.7 |
|  | No platinum | NR |  |  |  | 82.4 |
| HRD- | Platinum | NR | 2.30(0.72-7.60) | 0.15 |  | 78.4 |
|  | No platinum | NR |  |  |  | 88.6 |

**B DFS stratified by BRCA1/2 status and treatment**

|  |  | Median DFS, Month | HR(95%CI) | Log-rank *p* value | *P*-interaction | 5-year DFS,% |
| --- | --- | --- | --- | --- | --- | --- |
| BRCA^mut^ | Platinum | NR | 0.48(0.10-2.20) | 0.33 | 0.16 | 81.8 |
|  | No platinum | 116.8 |  |  |  | 78.9 |
| BRCA^wt^ | Platinum | NR | 1.50(0.55-4.10) | 0.42 |  | 85.5 |
|  | No platinum | NR |  |  |  | 88.2 |

**C DFS stratified by BRCA1/2 status**

|  | Median DFS, Month | HR(95%CI) | Log-rank *p* value |
| --- | --- | --- | --- |
| BRCA^mut^ | NR | 2.1(1.0-4.6) | 0.047 |
| BRCA^wt^ | NR |  |  |

Abbreviations: HRD, homologous recombination deficiency; DFS, Disease-Free Survival; NR, not reached

# Table S10. Univariable of Disease-Free Survival in the Platinum-Containing Group (Surgical Cohort).

| Characteristics | Level | Univariable analysis | |  | |
| --- | --- | --- | --- | --- | --- |
|  |  | Hazard ratio | 95% CI | *P* value | |
| HRD status | HRD-  (n=38) | Ref |  | 0.09 | |
|  | HRD+  (n=36) | 0.35 | 0.10-1.18 |  | |
| Age | <50  (n=42) | Ref |  | 0.76 | |
|  | ≥50  (n=32) | 1.19 | 0.40-3.54 |  | |
| BRCA 1/2 | Wild-type  (n=63) | Ref |  | 0.95 | |
|  | Mutant  (n=11) | 0.95 | 0.21-4.30 |  | |
| HRR-related genes | Wild-type  (n=44) | Ref |  | 0.69 | |
|  | Mutant  (n=30) | 1.25 | 0.42-3.72 |  | |
| Stage | I  (n=28) | Ref |  | 0.30 | |
|  | II-III  (n=46) | 1.98 | 0.54-7.27 |  | |
| Ki67 | <30  (n=14) | Ref |  | 0.67 | |
|  | ≥30  (n=58) | 0.76 | 0.20-2.80 |  | |
|  | | | | |  |
|  | | | | |  |

Abbreviations: A tumor was defined as HRD+ if having a HRD score of ≥30 and/or deleterious mutation in BRCA1/2, while a BRCA1/2-intact tumor with an HRD score of < 30 was defined as HRD-.; HRR, Homologous recombination repair.
